# The adequacy of aging techniques in vertebrates for rapid estimation of population mortality rates from age distributions

**DOI:** 10.1002/ece3.4854

**Published:** 2018-12-27

**Authors:** Meijuan Zhao, Chris A. J. Klaassen, Simeon Lisovski, Marcel Klaassen

**Affiliations:** ^1^ School of Life Sciences University of Science and Technology of China Hefei Anhui China; ^2^ Centre for Integrative Ecology, School of Life and Environmental Sciences Deakin University Geelong Victoria Australia; ^3^ Korteweg‐de Vries Institute for Mathematics University of Amsterdam Amsterdam The Netherlands; ^4^ Swiss Ornithological Institute Department of Bird Migration Sempach Switzerland

**Keywords:** age estimation, aging error, birth rate, demographics, mark–recapture, survival rate

## Abstract

As a key parameter in population dynamics, mortality rates are frequently estimated using mark–recapture data, which requires extensive, long‐term data sets. As a potential rapid alternative, we can measure variables correlated to age, allowing the compilation of population age distributions, from which mortality rates can be derived. However, most studies employing such techniques have ignored their inherent inaccuracy and have thereby failed to provide reliable mortality estimates. In this study, we present a general statistical model linking birth rate, mortality rate, and population age distributions. We next assessed the reliability and data needs (i.e., sample size) for estimating mortality rate of eight different aging techniques. The results revealed that for half of the aging techniques, correlations with age varied considerably, translating into highly variable accuracies when used to estimate mortality rate from age distributions. Telomere length is generally not sufficiently correlated to age to provide reliable mortality rate estimates. DNA methylation, signal‐joint T‐cell recombination excision circle (sjTREC), and racemization are generally more promising techniques to ultimately estimate mortality rate, if a sufficiently high sample size is available. Otolith ring counts, otolithometry, and age‐length keys in fish, and skeletochronology in reptiles, mammals, and amphibians, outperformed all other aging techniques and generated relatively accurate mortality rate estimation with a sample size that can be feasibly obtained. Provided the method chosen is minimizing and estimating the error in age estimation, it is possible to accurately estimate mortality rates from age distributions. The method therewith has the potential to estimate a critical, population dynamic parameter to inform conservation efforts within a limited time frame as opposed to mark–recapture analyses.

## 1 INTRODUCTION

In this time and era where many organisms are facing great environmental challenges, the need to evaluate the demographics of animal populations to guide conservation and management practices is high and rising. Mortality rates are among the most important demographic parameters for understanding population dynamics (Caughley, 1977). Probably, the most widely used methodologies to assess mortality rates in populations are mark–recapture analyses. However, mark–recapture analyses tend to require large and long‐term data sets. For instantaneous, snapshot estimation of mortality rates, age distributions within populations serve as an alternative and a series of models have been developed to that effect (Supporting information Appendix S1). A cited reference search for these models and refining by “age” in combination with “mortality” or “survival” in Web of Knowledge yielded 2065 studies (date accessed on 10 October 2018), implying the wide application of deducing mortality rate from age distribution. Although this approach bears notable promises for cases where urgent assessment of population status is required, there has thus far been little consideration of its accuracy involving a robust statistical approach.

Almost invariably the underlying age distributions used in the calculation of mortality rates are subject to error. Although various models assessed mortality rates from age distributions (Supporting information Appendix S1), all but Conn, Doherty, Nichols, Ricklefs, and Rohwer (2005) assumed that age was estimated without error. Thus, the reliability of deduced mortality rates was generally overrated. Conn et al. (2005) used only two age categories, that is, juvenile and adult, numerically investigating the consequences of misclassification to these two categories. Their study pointed out that substantial errors in mortality rate estimation can potentially be made using this approach even if other major assumptions, such as that the age distribution across the population under study is stable, are being met. Despite its critical importance, a robust statistical model to investigate the propagation of errors in age estimation to mortality rate estimates from age distributions has, to our knowledge, not previously been developed.

Birth and survival processes determine the age distribution within a population. The birth and survival distributions over time are therefore intimately linked with a population's age distribution. Provided that the age distribution of a population can be measured, and the birth distribution of that population is known, the survival distribution can be estimated, and vice versa. Birth distributions can often be assessed with much more ease than survival distributions or can alternatively be reasonably assumed (e.g., a uniform distribution; that is, a constant average birth rate; Ball, Britton, & Trapman, 2017; Thornley & France, 2016). The interest therefore primarily lies with being able to reliably estimate a survival distribution from a population's age distribution.

Our study has three aims that are being addressed in separate sections of the paper: (a) To provide a detailed statistical description of the relationship between a population's birth rate, survival rate, and age distributions, we present a statistical model for the analytical derivation of mortality rates and their confidence intervals from age distributions, taking errors in age estimation into account. (b) To evaluate the accuracy of different aging techniques in determining age, we next reviewed the accuracy of a range of popular or potentially promising aging techniques employed in vertebrates. These include measurement of telomere length, DNA methylation, signal‐joint T‐cell recombination excision circle (sjTREC), racemization, otolith ring counts, otolithometry (measurement of otolith mass, length, width, and height), age‐length keys, and skeletochronology. (c) Finally, we used our statistical model to evaluate the suitability of these aging techniques to accurately estimate mortality rates.

## 2 MATERIALS AND METHODS

### 2.1 Model description

We here provide a brief outline of the statistical models that are presented in more detail, including all assumptions made, in Supporting information Appendix S2. To facilitate cross‐referencing between the below and Supporting information Appendix S2, we retain equation numbers as used is Supporting information Appendix S2.

Consider a well‐defined population for which we want to evaluate its dynamics and potentially simulate or predict its future development. The development of this population is necessarily stochastic and is determined by the survival function $1-F_{S}\left(s\right)$ and the birth time density function $f_{T}\left(t\right)$ of its members, ultimately resulting in the population's age density function $f_{Y}\left(y\right)$. Here, the subscripts *S, T* and *Y* denote the survival time, the time of birth, and the age, respectively, of a random individual from the population. Furthermore, we follow the convention of indicating distribution functions by *F* and probability density functions by *f.* The relationship between these three entities, survival time, time of birth, and age, is given by formula

(1) $$f_{Y}\left(y\right)=\frac{\left(1-F_{S}\left(y\right)\right)f_{T}\left(-y\right)}{\int_{0}^{\infty }\left(1-F_{S}\left(t\right)\right)f_{T}\left(-t\right)dt},\;-\infty <y<\infty ,$$

or equivalently.

(2) $$1-F_{S}\left(s\right)=\frac{f_{T}\left(0\right)f_{Y}\left(s\right)}{f_{T}\left(-s\right)f_{Y}\left(0\right)},-\infty <s<\infty .$$

Given two of these three entities, the third one is determined. In the majority of cases, $f_{T}\left(t\right)$ can be assessed in some way or reasonably assumed. Consequently, an estimate of the survival function $1-F_{S}\left(s\right),$ which is of key interest, can be computed via Equation (), provided $f_{Y}\left(y\right)$ can be estimated.

To obtain an estimate of $f_{Y}\left(y\right),$ one has to measure the age of individuals from a population directly. However, this is almost always impossible and one consequently has to rely on measuring other variables, so‐called age proxies *X*, that correlate strongly with age *Y*. In Supporting information Appendix S2, statistical solutions are provided for the case where a single (*d* = 1) or multiple (*d* > 1) age proxies *X* are described as a function of age *Y*. In the framework of the current study where we wish to review the suitability of specific age proxies in isolation, we specifically consider the case *d *= 1 with *X* = *X* ^(1)^, a one‐dimensional age proxy. For *d* = 1, we assume that there exists a strictly monotone, known function *g*(*y*), a known positive constant *σ*, and a random variable ε with known density $f_{\varepsilon }\left(z\right),$ distribution function $F_{\varepsilon }\left(z\right),$ mean Eε=0, and variance $E\varepsilon^{2}=1,$ such that

(3) $$X=g\left(Y\right)+\sigma \varepsilon$$

which ultimately (see Supporting information Appendix S2) yields:

(4) $$f_{X}\left(x\right)=\int_{0}^{\infty }\frac{1}{\sigma }f_{\varepsilon }\left(\frac{x-g\left(Y\right)}{\sigma }\right)f_{Y}\left(y\right)dy.$$

Furthermore, for the specific aims of our study we make the classic assumption that the monotone function $g\left(y\right)$ is linear, that is, that there exist known constants *α* and *β* with

(5) $$g\left(y\right)=\alpha +\beta y,\;y>0.$$

Thus, the dependence of the age proxy *X* on the age *Y* is described via linear regression with normal error.

As outlined in Supporting information Appendix S2, we need not necessarily make any assumptions on the class of survival functions $1-F_{S}\left(s\right)$ and resort to a so‐called nonparametric estimation problem to estimate the density function $f_{X}\left(x\right)$. However, in such cases estimating the density function $f_{Y}\left(y\right)$ is a so‐called deconvolution problem, which is notoriously hard to solve. For these problems, convergence rates in *n* (i.e., the number of age proxies measured) are extremely slow, like $\sqrt{\text{ln}n}$; see Carroll and Hall (1988). On the other hand, if restriction of the survival function to a parametric class of survival functions is justified, estimation of the survival function at a $\sqrt{n}$ rate becomes feasible.

To satisfy the wish for a parametric class of survival functions, we may consider an exponential distribution for the survival time S of a newborn (chosen at random) from a population. This means that we assume the existence of a positive number λ with.

(6) $$1-F_{S,\lambda }\left(s\right)=P\left(S>s\right)=e^{-\lambda s}={\left(1-m\right)}^{s},\;s\geq 0,$$

and $\lambda =-\text{ln}\left(1-m\right),$ which implies that the density of S satisfies

(7) $$f_{S,\lambda }\left(s\right)=\lambda e^{-\lambda s},\;s\geq 0.$$

Loosely speaking, *m* is the probability that an individual dies within the next time unit. Therefore, *m* is called the mortality rate and 0<m<1 holds.

Although this simplification, which assumes a constant mortality rate *m* with age, is not strictly necessary from a mathematical perspective (i.e., models allowing for much more general or complex relationships between survival and age, and thus mortality rate and age, are feasible as outlined above and in Section 3 of Supporting information Appendix S2), it dramatically reduces the required sample size. In many cases, this is also a reasonable assumption for two reasons. Firstly, some species do show either a constant mortality rate or a roughly constant mortality rate before reaching a very advanced age (6 out of 23 vertebrates investigated in Jones et al., 2014). Secondly, although in the remaining species the relationship between age and mortality rate varies, it often increases exponentially with age, resulting in few individuals within wildlife populations living long enough to be impacted by a substantially increased mortality rate with age. Hence, estimating age‐specific mortality rates often require large, long‐term data sets, be it mark–recapture data or population age distribution data. Such data are, however, available for very few species only (Jones et al., 2014). Most case studies lack sufficient data and thus estimate mortality rate based on the assumption of a constant adult mortality. Given the relatively low number of individuals affected by advanced‐age‐related changes in mortality, the potential impact on the population dynamics of such assumption may also be limited. Finally, this simplification is notably warranted in the present case, where the aim of the exercise is primarily focused on evaluating the suitability of a range of popular or potentially promising vertebrate aging techniques to establish population age distributions with the purpose of estimating survival functions.

Next to the value of the age proxy *X* of sampled individuals, one might be able to observe additional information about these individuals, like their sex and other characteristics. In principle, such covariates might help in obtaining a more precise estimate of the true age *Y*. However, given the limited availability of such data in the literature, we will not pursue this approach any further in this paper.

To simplify further, we assume that the population is completely stable, in the sense that $f_{T}\left(t\right)$ is the uniform density on [−τ,0] with τ tending to infinity. In view of Equations () and (), we have

(8) $$f_{Y,\lambda }\left(y\right)=\frac{1-F_{S,\lambda }\left(y\right)}{\int_{0}^{\infty }\left(1-F_{S,\lambda }\left(t\right)\right)dt}=\frac{e^{-\lambda y}}{1/\lambda }=\lambda e^{-\lambda y},\;y\geq 0.$$

This means that the age *Y* of an arbitrary individual from the population has the same exponential distribution as the survival time *S* of a newborn. Next, combination of Equations (10) and (18) shows that the density of the age proxy *X* becomes.

(9) $$f_{X,\lambda }\left(x\right)=\int_{0}^{\infty }\frac{1}{\sigma }\varphi \left(\frac{x-\alpha -\beta y}{\sigma }\right)\lambda e^{-\lambda y}dy,\;x\in R.$$

Writing $\mu =\sigma \lambda /\left|\beta \right|$ we note that estimation of the mortality rate

(10) $$m=1-\text{exp}\left(-\lambda \right)=1-\text{exp}\left(-\left|\beta \right|\mu /\sigma \right)$$

is equivalent to estimation of the parameter μ, which we propose to call proxy coefficient. With $\Phi \left(z\right)$ denoting the standard normal distribution function, Equation (20) can be rewritten in terms of μ as

(11) $$f_{X,\lambda }\left(x\right)=\frac{\mu }{\sigma }\exp\left(\frac{1}{2}\mu^{2}-\mu \frac{\beta }{\left|\beta \right|}\frac{x-\alpha }{\sigma }\right)\Phi \left(\frac{\beta }{\left|\beta \right|}\frac{x-\alpha }{\sigma }-\mu \right).$$

The correlation between the age Y and the age proxy X equals 1/$\sqrt{1+\mu^{2}.}$ So, the smaller the proxy coefficient μ, the larger the correlation between the age and the age proxy.

To estimate the value of m or equivalently of $\mu =-\frac{\sigma \left(\ln\left(1-m\right)\right)}{\left|\beta \right|}$ (cf. Equation ), we take a random sample of size n from the population and determine the values of age‐correlated variable X of the selected individuals. Let us denote these values as $x_{1},\ldots ,x_{n}$, which are viewed as realizations of the independent random variables $X_{1},\ldots ,X_{n}$, with density from Equation (23).

In Supporting information Appendix S2, it is shown that the Fisher information for proxy coefficient μequals.

(12) $$J\left(\mu \right)=\frac{1}{\mu^{2}}-1-\mu^{2}+\mu \int_{-\infty }^{\infty }\frac{\varphi \left(z\right)}{\Phi \left(z\right)}\varphi \left(z+\mu \right)dz$$

and that the asymptotically efficient estimator ${\hat{\mu }}_{n}$ of μ can be defined as

(13) $${\hat{\mu }}_{n}=\left(1+\frac{1}{{\hat{J}}_{n}}\right){\overset{¯}{\mu }}_{n}-\frac{1}{n{\hat{J}}_{n}}\sum_{i=1}^{n}\frac{\varphi }{\Phi }\left(\frac{\beta }{\left|\beta \right|}\frac{X_{i}-\alpha }{\sigma }-{\overset{¯}{\mu }}_{n}\right)$$

with φ/$\Phi \left(z\right)$ written as short hand for φ(*z*)/$\Phi \left(z\right).$ “Asymptotically efficient” means that the estimator has approximately a normal distribution for large sample sizes n with the true value μ as mean and the smallest possible variance, namely $\frac{1}{nJ\left(\mu \right)}.$

In view of Equations (15) and (22), the corresponding efficient estimator ${\hat{m}}_{n}$ for m equals.

(14) $${\hat{m}}_{n}=1-\exp\left(-\frac{\left|\beta \right|{\hat{\mu }}_{n}}{\sigma }\right).$$

By taking many samples of size n from the set of numbers {$x_{1},\ldots ,x_{n}$}, computing the corresponding estimates of m, and estimating in this way the distribution of ${\hat{m}}_{n},$we may construct a bootstrap confidence interval for m.

As an alternative 95% confidence interval for m, we mention.

(15) $$\left[{\hat{m}}_{n}-\frac{1.96}{\sqrt{nI\left({\hat{m}}_{n}\right)}},\;{\hat{m}}_{n}+\frac{1.96}{\sqrt{nI\left({\hat{m}}_{n}\right)}}\right],$$

where the function.

(16) $$I\left(m\right)=\frac{\sigma^{2}J\left(-\frac{\sigma }{\left|\beta \right|}\ln\left(1-m\right)\right)}{\beta^{2}{\left(1-m\right)}^{2}}$$

is evaluated at ${\hat{m}}_{n}.$Here, $J\left(\mu \right)$is as in Equation (32) and $I\left(m\right)$is the Fisher information for m. The 95% confidence range (CR) may be calculated by deducting the lower limit from the upper limit and equals

(17) $$\text{CR}\left(95\right)=\frac{3.92}{\sqrt{nI\left({\hat{m}}_{n}\right)}}.$$

We define the empirical 95% error percentage as.

(18) $$\text{EEP}\left(95\right)=\frac{\text{CR}\left(95\right)}{{\hat{m}}_{n}}\times 100\%=\frac{392}{\sqrt{n}}\times \frac{1}{{\hat{m}}_{n}\sqrt{I\left({\hat{m}}_{n}\right)}}\%,$$

which estimates the theoretical 95% error percentage.

(19) $$\text{EP}\left(95\right)=\frac{392}{\sqrt{n}}\times \frac{1}{m\sqrt{I\left(m\right)}}\%,$$

with m the true value of the mortality rate. We used EEP(95) to assess the accuracy of mortality rate estimation from age distributions with large values indicating high variation and thus lower accuracy.

### 2.2 Review of aging techniques

To obtain a general impression of the performance of each aging technique when assessing mortality rate using age distributions, we reviewed eight aging techniques for vertebrates including measurement of telomere length, DNA methylation, sjTREC, racemization, otolith ring count, otolithometry, age‐length keys, and skeletochronology. These eight aging techniques were reviewed because they are well‐known indicators that to some extent correlate with age (i.e., telomere length, otolith ring count, otolithometry, age‐length keys, and skeletochronology) or are promising indicators in determining age with low errors (i.e., DNA methylation, sjTREC, and racemization). We reviewed these techniques in terms of the animal classes to which they have been applied, their accuracy in predicting age and factors other than age (e.g., environmental factors, health status) influencing their suitability as age estimators (background details of the eight aging techniques are presented in Supporting information Appendix S3). To evaluate their accuracy as age predictors in vertebrates, we selected 218 data points from 123 case studies, extracted the relationship between age estimators and true age, and obtainedβ/σ the crucial indicator for the variation in estimated mortality rate (see Equations and ) and the $R^{2}$ for each study (data are presented in Supporting information Appendix S4). Specifically, we conducted a Web of Knowledge search using the key words “age estimation” or “age determination” or “age assessment” or “estimat* age” or “assign* age” combined with the name of each aging technique. From these studies and the relevant studies cited therein, we selected only those investigating long‐lived (maximum age estimated ≥2 years) vertebrates, with a sample size larger than five, including both laboratory‐ and field‐based studies. Moreover, we excluded cases investigating the correlation of age proxy and real age in diseased individuals. Only a small proportion of these studies provided raw data that we could directly use to calculate $R^{2}$ and β/σ. In the remaining cases where only data distributions were provided, we used bootstrapping over 10,000 iterations to generate data and subsequently calculate a mean $R^{2}$ and β/σ. To evaluate the performance of the various aging techniques within animal classes, for each of these groups the median and range of $R^{2}$ and β/σ were calculated.

**Figure 1:**
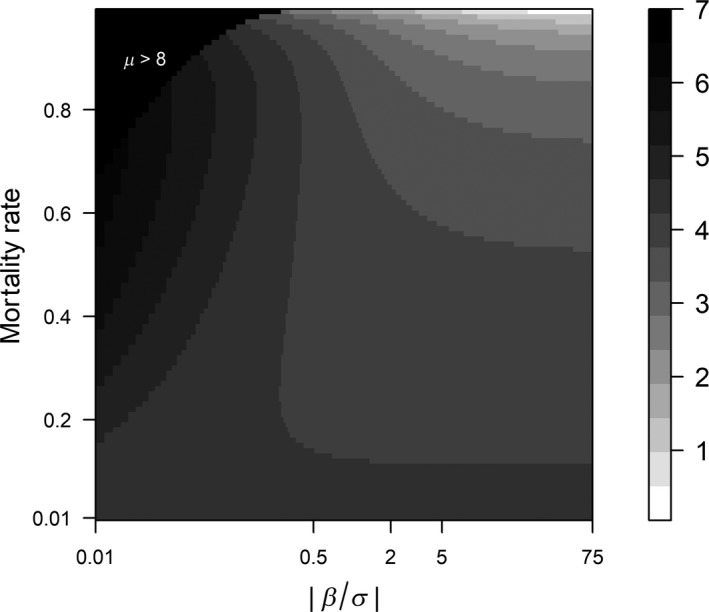
Lookup plot for the basic factor in the error percentage. Lookup plot for $\frac{1}{m\sqrt{I\left(m\right)}}$, the basic factor in the error percentage EP(95) (calculated as the 95% confidence range divided by the expected mortality rate *m* using Equation for various combinations of mortality rate (*m*;* y*‐axis) and aging parameter ($\left|\beta /\sigma \right|$; *x*‐axis). β and σ represent the slope and standard deviation of the error in a linear regression of age against age markers estimated by different aging techniques, respectively. The absolute value of β/σ, $\left|\beta /\sigma \right|$, is the key variable representing the accuracy of aging techniques in the estimation of age and thus the estimation of mortality rate. Shadings range from black (poor performance) via gray to white (good performance), where black shading on the left‐hand corner indicates combinations of *m* and $\left|\beta /\sigma \right|$ which yield proxy coefficient *μ* > 8. For such cases, $\frac{1}{m\sqrt{I\left(m\right)}}$ is large and the computation of its exact value is unreliable. We thus considered techniques yielding low $\left|\beta /\sigma \right|$ values that result in *μ* > 8 as performing very poorly and unable to accurately estimate mortality rate.

To evaluate the performance of the reviewed aging techniques when assessing mortality rate using age distributions, we constructed a lookup plot (Figure 1) for $\frac{1}{m\sqrt{I\left(m\right)}},$the basic factor in the 95% error percentage EP(95) (see Equation (44) with the absolute value of β/σ or $\left|\beta /\sigma \right|$ ranging from 0.01 to 75 (range of observed $\left|\beta /\sigma \right|$, the upper limit 75 is the second largest observed value of$\left|\beta /\sigma \right|$, the largest value]]> infinite) and the mortality rate m ranging from 0.01 to 0.99. Using Equation (44), we also constructed a lookup table (Table 1) to assist determining the minimum sample size n required to estimate mortality rate with a specific accuracy (i.e., with a desired 95% error percentage EP(95)) for various combinations of $\left|\beta /\sigma \right|$ and expected mortality rate m.

**Table 1:** The minimum number of individuals required to estimate mortality rate with a specific accuracy (i.e., the 95% error percentage EP (95)) for various combinations of $\left|\beta /\sigma \right|$ and expected mortality rate *m*

	*m*	|*β*/*σ*|						
		0.1	0.2	0.5	1	2	5	10
EP (95) = 5%	0.1	9,813	6,682	5,738	5,584	5,542	5,530	5,528
	0.2	23,431	9,150	5,650	5,105	4,953	4,907	4,900
	0.3	51,048	14,055	5,821	4,690	4,376	4,278	4,263
	0.4	90,927	22,168	6,244	4,321	3,806	3,644	3,618
	0.5	139,059	33,378	6,935	3,981	3,238	3,005	2,967
EP (95) = 10%	0.1	2,454	1671	1,435	1,396	1,386	1,383	1,382
	0.2	5,858	2,288	1,413	1,277	1,239	1,227	1,225
	0.3	12,762	3,514	1,456	1,173	1,094	1,070	1,066
	0.4	22,732	5,542	1561	1,081	952	911	905
	0.5	34,765	8,345	1734	996	810	752	742
EP (95) = 20%	0.1	614	418	359	349	347	346	346
	0.2	1,465	572	354	320	310	307	307
	0.3	3,191	879	364	294	274	268	267
	0.4	5,683	1,386	391	271	238	228	227
	0.5	8,692	2087	434	249	203	188	186
EP (95) = 30%	0.1	273	186	160	156	154	154	154
	0.2	651	255	157	142	138	137	137
	0.3	1,418	391	162	131	122	119	119
	0.4	2,526	616	174	121	106	102	101
	0.5	3,863	928	193	111	90	84	83
EP (95) = 40%	0.1	154	105	90	88	87	87	87
	0.2	367	143	89	80	78	77	77
	0.3	798	220	91	74	69	67	67
	0.4	1,421	347	98	68	60	57	57
	0.5	2,173	522	109	63	51	47	47

Notes

β and σ represent the slope and the standard deviation of the error in a linear regression of age against age markers estimatedy different aging techniques, respectively. The absolute value of β/σ, $\left|\beta /\sigma \right|$,is the key variable representing the accuracy of aging techniques in the estimation of age and thus in the estimation of mortality rate.

## 3 RESULTS

In our statistical approach, the accuracy of the aging technique $\left|\beta /\sigma \right|$, the sample size n, and the expected mortality rate m are the three determinant parameters for the variation in the estimated mortality rate. In Figure 1, we translated the simultaneous variations in $\left|\beta /\sigma \right|$ and m into the variation in the basic factor (i.e., $\frac{1}{m\sqrt{I\left(m\right)}}$) that correlates positively with the error percentage EP(95) (Equation ). This factor, and thus EP(95), generally decreases with an increase in $\left|\beta /\sigma \right|$. However, in the range of 0.2 <$\left|\beta /\sigma \right|$<2, the basic factor decreases rapidly after which the basic factor changes little for $\left|\beta /\sigma \right|$>2, plateauing around $\left|\beta /\sigma \right|$= 5.

An interaction exists between $\left|\beta /\sigma \right|$and m. When $\left|\beta /\sigma \right|$>0.5, the basic factor generally decreases with an increase in m *,* meaning that for a certain sample size the accuracy of an aging technique should increase when m decreases in order to keep $EP\left(95\right)$ stable. However, when $\left|\beta /\sigma \right|$ <0.5, the basic factor and thus EP(95) increase rather than decrease with m. When mortality rate m tends to be high, and $\left|\beta /\sigma \right|$ tends to be low, the μ in Equation (22) is high. For large μ values, for example, μ>8, the basic factor in EP(95) is high, but the computation of its exact value is numerically unreliable, due to the division of two extremely small numbers in element $\mu \int_{-\infty }^{\infty }\frac{\phi \left(z\right)}{\Phi \left(z\right)}\phi \left(z+\mu \right)dz$ in Equation (i.e., in the computation of *J*, which is subsequently used in Equations and ). We thus considered aging techniques yielding low $\left|\beta /\sigma \right|$ values that result in *μ* > 8 as performing very poorly and unable to accurately estimate mortality rate. We like to stress that Equation (44) and hence Figure 1 and its properties sketched here are consequences of the model we have chosen via Equations (15) and (19).

Table 1 shows the minimum sample size n needed for a sufficiently accurate estimate of mortality rate. When the aging technique performs poorly, that is,$\left|\beta /\sigma \right|$ is low, the required sample size n to achieve a low EP(95) is large. For instance, more than 400 individuals are required to achieve an EP(95) of 20% when $\left|\beta /\sigma \right|$ < 0.2. Whether or not such a high sample size is difficult to achieve very much depends on the ease of obtaining animals for aging, which may be species and context dependent, and the (costs of the) methods involved in estimating age. Yet, it is clear that one should generally strive for the use of aging techniques with $\left|\beta /\sigma \right|$>0.2 when estimating mortality rate is one of the ultimate targets.

The eight aging techniques achieved highly different accuracies in terms of their $R^{2}$ and $\left|\beta /\sigma \right|$ (Table 2). Telomere length invariably yielded poor matches. On average, DNA methylation, sjTREC, and racemization performed slightly better. Otolithometry, age‐length keys, otolith ring count, and skeletochronology tended to provide the best outcomes. However, all aging techniques showed markedly different performances across taxa and studies with even the most promising techniques, otolith ring count and skeletochronology, occasionally proved to be unreliable.

**Table 2:** Summary of accuracy in aging across studies employing eight different aging techniques for a range of vertebrate taxa

	*N*	*R* ^2^	|*β*/*σ*|
Telomere length			
Human	14	0.30 (0.09–0.69)	0.03 (0.01–0.08)
Mammal	8	0.33 (0.02–0.64)	0.10 (0.03–0.42)
Bird	20	0.34 (0.00–0.82)	0.10 (0.00–1.60)
Fish	18	0.29 (0.00–0.85)	0.50 (0.01–1.02)
Shark	2	0.06 (0.03–0.10)	0.04 (0.04–0.05)
Reptile	6	0.05 (0.00–0.73)	0.02 (0.01–0.46)
DNA methylation			
Human	9	0.82 (0.06–0.98)	0.15 (0.02–0.26)
Mammal	4	0.75 (0.00–0.93)	0.24 (0.00–0.37)
sjTREC			
Human	6	0.76 (0.65–0.85)	0.47 (0.10–2.38)
Mammal	1	0.00 (0.00–0.00)	0.00 (0.00–0.00)
Racemization			
Human	54	0.96 (0.55–0.99)	0.32 (0.06–2.39)
Mammal	2	0.92 (0.90–0.93)	0.38 (0.36–0.39)
Bird	3	0.45 (0.02–0.53)	0.17 (0.04–0.98)
Otolithometry			
Fish	37	0.75 (0.28–0.92)	1.81 (0.19–75.20)
Age‐length keys			
Fish	18	0.78 (0.30–0.92)	1.89 (0.23–12.58)
Otolith ring count			
Fish	8	0.92 (0.60–1.00)	2.42 (1.27–Inf)
Skeletochronology			
Mammal	1	1.00 (1.00–1.00)	4.80 (4.80–4.80)
Reptile	6	0.96 (0.00–1.00)	1.86 (0.00–Inf)
Amphibian	1	1.00 (1.00–1.00)	Inf (Inf–Inf)

*N*, number of case studies; *R* ^2^, β,and σ represent the coefficient of determination, the slope, and the standard deviation of the error in a linear regression of age against age markers estimated by different aging techniques, respectively. The absolute value of β/σ, $\left|\beta /\sigma \right|$,is the key variable representing the accuracy of aging techniques in the estimation of age and thus in the estimation of mortality rate. Humans considered separate from other mammals. *N*, median, and range for $R^{2}$ and $\left|\beta /\sigma \right|$ are provided for each taxon. Individual data for all case studies are provided in Supporting information Appendix S4.

sjTREC means signal‐joint T‐cell recombination excision circles; accuracies of otolithometry and age‐length keys are probably inflated since these used otolith ring counts to assess true age without validation; Inf indicates infinite $\left|\beta /\sigma \right|$ for *σ* = 0, that is, no error in age determination.

Although showing a relatively good correlation with age in some fish studies, telomere length seems to be a generally poor proxy for age. In many studies, telomere length did not correlate with age and the median $R^{2}$ across taxa was <0.35. With the exception of fishes, in most cases $\left|\beta /\sigma \right|$ < 0.2 (Table 2), indicating that the method is not sufficiently adequate to estimate mortality rate from age distributions.

DNA methylation performed slightly better than telomere length, with a median *R* ^2^ across taxa between 0.75 and 0.85. Although the median $\left|\beta /\sigma \right|$ in mammals was just above 0.2, $\left|\beta /\sigma \right|$ varied considerably across studies and was incidentally as low as 0 (Table 2).

sjTREC in humans performed slightly better than DNA methylation, with a median $\left|\beta /\sigma \right|$ of 0.47 (Table 2). However, the single application of sjTREC in mammals showed no correlation with age (Ito, Yoshimura, & Momoi, 2015).

**Figure 2:**
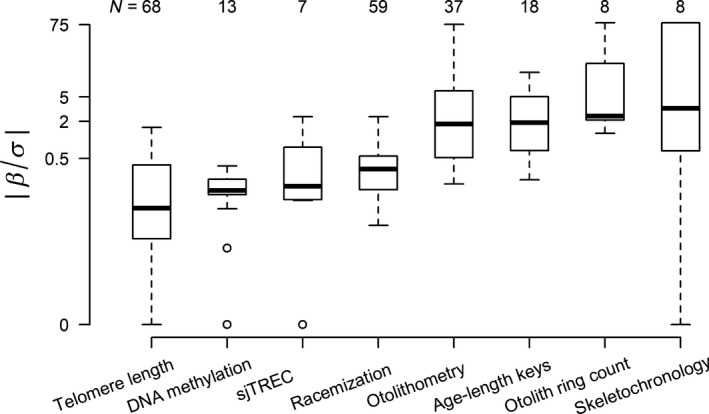
Performance of the reviewed aging techniques. Box plots displaying the variations within and among the reviewed aging techniques (Supporting information Appendix S3) in their correlations with age in terms of $\left|\beta /\sigma \right|$. β and σ represent the slope and the standard deviation of the error in a linear regression of age against age markers estimated by different aging techniques, respectively. The absolute value of β/σ, $\left|\beta /\sigma \right|$, is the key variable representing the accuracy of aging techniques in the estimation of age and thus in the estimation of mortality rate. Aging techniques along the *x*‐axis are ranked in order of increasing median $\left|\beta /\sigma \right|$ across case studies. Numbers along the top of the panel denote number of case studies. The thick line within each box and whisker plot represents the median, and the lower and upper box border represent the first and the third quartile, respectively. Whiskers denote the lower and upper 95% confidence interval. Dots outside the whiskers are outliers above or below the 95% confidence interval.

Racemization performed similarly to DNA methylation and sjTREC with considerable variation within and between taxa. A number of racemization studies in humans and mammals achieved a median *R* ^2^ > 0.9 and a median$\left|\beta /\sigma \right|$ > 0.2. The *R* ^2^ and $\left|\beta /\sigma \right|$ of individual studies, however, could be as low as those found in studies employing telomere length (Figure 2 and Table 2). Moreover, to date its application to nonhuman vertebrates has been highly limited (Table 2).

Otolith ring counts, which are limited to studies in fish, showed best correlations with age, with a median *R* ^2^ and $\left|\beta /\sigma \right|$ of 0.9 and 2.4, respectively (Table 2). This is therewith the only technique achieving a median $\left|\beta /\sigma \right|$ higher than 2. One study even scored a 100% accuracy (Gooley, 1992). Both otolithometry and age‐length keys, for which we found studies in fish exclusively although indeterminate growth is also present in reptiles and amphibians, could likewise provide good estimates of age, with $\left|\beta /\sigma \right|$ close to 2. It should be noted that in all these studies where age was based on otolith ring counts, this age was considered to be the true age and that our estimates of accuracy for these studies are thus potentially inflated.

Albeit sample size was limited to one in both taxa, the (near) 100% accuracy for skeletochronology in amphibians (Friedl & Klump, 1997) and mammals (Castanet et al., 2004) indicates great promise for this technique (Table 2). This promise was supported by the larger sample of reptile studies yielding |*β/σ*| close to 2 (Table 2).

## 4 DISCUSSION

Despite their wide use to this effect, half of the aging techniques reviewed were not sufficiently reliable to invariably yield satisfyingly accurate mortality rate estimates, for populations with a roughly constant mortality rate during adulthood. Telomere length performed worst; the two molecular methods, DNA methylation and sjTREC, and the chemical method, racemization, perform slightly better. The two techniques that can exclusively be used in fish (i.e., otolith ring count and otolithometry) and two techniques with potential wider application, that is, age‐length keys and skeletochronology, performed best. If we would consider error percentages ≤20% at an assumed mortality rate of the population of 0.3 as sufficiently accurate, $\left|\beta /\sigma \right|$ would have to be higher than 0.34 when n = 500. Under this scenario, only 31% of the case studies using telomere length resulted in sufficiently accurate mortality rate estimates. This proportion increased to 62% in studies using DNA methylation, sjTREC, or racemization; 75% in skeletochronology; and increased to 92% in studies using otolith ring count, otolithometry, or age‐length keys. Thus, any application of these aging techniques for mortality rate estimates would need to carefully evaluate the error in age estimation and should not take high accuracies in age estimation for granted. Finally, one could also resort to measuring and combining multiple age proxies to estimate age and therewith reduce error and improve accuracy. In Supporting information Appendix S2 Section 2, the statistical basis for this approach is provided.

Although not explicitly addressed in our review since the examples are few, we noted that the performances of some aging techniques varied considerably between populations, genders, and tissues examined, for example, between populations using otolithometry (Newman, 2002), genders using age‐length keys (Ordines, Valls, & Gouraguine, 2012), and tissues using telomere length (Izzo, 2010). Thus, in addition to using multiple age proxies, the inclusion of covariates in the estimation of age from age proxies may be an additional avenue to improve accuracy.

Telomere length has been one of the first molecular indicators that were found to correlate with age. However, it was far from satisfying to allow for its use in “mortality rate from age distribution modeling,” consistent to the conclusion by Dunshea et al. (2011) that telomere length is insufficiently accurate to estimate age in vertebrates. The aging techniques here reviewed are among the most promising or popular ones. In fish, growth ring counts in other hard structures, such as scales, vertebrae, and fins, have also been employed in age estimation but have generally been found to be less accurate than otolith ring counts (e.g., Vandergoot, Bur, & Powell, 2008, Ma, Xie, Huo, Yang, & Li, 2011). Other morphological measurements and molecular biomarkers have also been used in vertebrates. For instance, measurement of shoulder height in elephant (Shrader et al., 2006); pentosidine in many mammalian species (Brownlee, Vlassara, Kooney, Ulrich, & Cerami, 1986; Sell et al., 1996) and poultry (Iqbal, Probert, & Klandorf, 1997); and lipofuscin in fishes (Girven, Gauldie, Czochanska, & Woolhouse, 1993) and humans (Goyal, 1982). However, all these techniques were less accurate than the reviewed alternatives, especially at older age, and were thus not included in our review.

Besides the accuracy of aging techniques in terms of $\left|\beta /\sigma \right|$, sampling effort also plays a key role in the accuracy of mortality estimates. For populations with an expected m lower than 0.5, to obtain a mortality estimate with an error percentage under 20%, one has to sample a large number of individuals, for example, >400 when $\left|\beta /\sigma \right|$ is below 0.2. Sampling effort under 180 individuals in this case can never achieve such proposed accuracy. Therefore, a combination of a reliable aging technique and a large sample size is crucial to obtain an accurate mortality estimate.

Although estimation of mortality rate from population age distributions has been widely applied, our model is the first to assess the accuracy of age distribution‐based mortality rate by taking errors in aging into consideration using a robust statistical model. The model makes a set of assumptions (see Section 4 of Supporting information Appendix S2) of which two are particularly noteworthy: (1) that the population is stable in the survival function of its individuals and (2) that mortality rate is constant across age groups. Fortunately, for assumption (1) to hold, the population need not be stable in numbers, but only in its age distribution. Nevertheless, we highlight that it should be realized that such age distributions are the result of population dynamic processes over time, including variations therein. The model assumes that such variations do not exist. If these variations do exist, as in wild populations, it will not detriment the performance of the model if those variations distribute around a mean and do not vary with age or time; such possibilities need to be considered in the interpretation of the model's outcomes. As outlined in *Materials and methods* and Supporting information Appendix S2, assumption 2 is not a prerequisite to allow estimation of mortality rates from age distributions taking errors in age proxies into consideration. However, assuming a constant mortality rate across age groups greatly reduces sample size requirements (to $\sqrt{n}$; Supporting information Appendix S2), which is of great importance considering the still large sample sizes required using this simplifying assumption. Admittedly, a constant mortality rate with age does not conform with reality in a range of species. For vertebrates where this has been investigated in detail, it applied to 6 out of 23 species (Jones et al., 2014). Nevertheless, it is an importantly simplifying assumption that is frequently adopted in initial analyses of population dynamics, especially for populations under threat with a lack of historical data to derive an age‐specific mortality rate. Moreover, depending on the nature of mortality–age relationships, of which many different forms exist (Jones et al., 2014), deviations from a roughly constant mortality rate with age may only occur at a highly advanced age and apply to a small, potentially negligible fraction of the population only. Such deviations from constant mortality rate may also hold for immatures, which in many species can be easily identified and removed from the analysis. Our statistical model thus serves as a first step before considering more complex alternatives, which will either require considerably larger data sets or, if resorting to mark–recapture, large longitudinal data sets. Irrespectively, we emphasize that while our specific model does not account for potential differential mortality rates with age, it still provides a good impression on how errors in aging translate into errors in mortality rate estimation.

The specific model here presented is applicable to any proxy for age that has, or can be converted to, a linear relationship with age. The application of the model to the reviewed data highlights that a relatively accurate aging technique and large sampling effort are needed to yield reliable mortality rate estimates. However, as opposed to mark–recapture studies, the method still has great potential for conservation efforts where time is at a premium, allowing the estimation of mortality rate as a crucial population dynamic parameter within a limited period of time.

## CONFLICT OF INTEREST

None declared.

## AUTHOR CONTRIBUTION

MK and MZ conceived the ideas; CK, MK, and MZ designed methodology; MZ collected the data; MZ and SL analyzed the data; MZ and MK led the writing of the main manuscript; and CK led the writing of Supporting information Appendix S2. All authors contributed critically to the drafts and gave final approval for publication.

## Data Availability

*R* ^2^ and |*β*/*σ*| for case studies employing eight different aging techniques will be archived in *Dryad Digital Repository* should the manuscript be accepted.
