# Supplementary material for: The adequacy of aging techniques in vertebrates for rapid estimation of population mortality rates from age distributions
Source: Ecol Evol. 2018 Dec 27;9(3):1394–402. doi: 10.1002/ece3.4854 (PMC6374686; doi:10.1002/ece3.4854)
Supplement: Supplementary file 1 [file ECE3-9-1394-s001.docx]

**Appendix S1. Models deducing mortality rate from age distributions.**

| **Source** | **Age-categories** | **Errors included?** |
| --- | --- | --- |
| ([Gompertz, 1825](#_ENREF_4)) | 0,1,...max | N |
| ([Makeham, 1860](#_ENREF_7)) | 0,1,...max | N |
| ([Witten, 1987](#_ENREF_14)) | 0,1,...max | N |
| ([Ricker, 1975](#_ENREF_8)) | 0,1,...max | N |
| ([Siler, 1979](#_ENREF_11)) | 0,1,...max | N |
| ([Heligman and Pollard, 1980](#_ENREF_6)) | 0,1,...max | N |
| ([Finch, 1990](#_ENREF_3)) | 0,1,...max | N |
| ([Udevitz and Ballachey, 1998](#_ENREF_12)) | 0,1,...max | N |
| ([Udevitz and Gogan, 2012](#_ENREF_13)) | 0,1,...max | N |
| ([Ferreira and Van Aarde, 2008](#_ENREF_2)) | Four | N |
| ([Sibly et al., 1997](#_ENREF_10)) | Three | N |
| ([Ricklefs, 1997](#_ENREF_9)) | Two | N |
| ([Green, 2004](#_ENREF_5)) | Two | N |
| ([Conn et al., 2005](#_ENREF_1)) | Two | Y |

Depicted are the number of age categories used as well as whether errors in age estimation were considered in the estimation of mortality rate.

**References**

CONN, P. B., DOHERTY, P. F., NICHOLS, J. D., RICKLEFS, R. E. & ROHWER, S. 2005. Comparative demography of New World populations of thrushes (*Turdus spp.*): Comment. *Ecology,* 86**,** 2536-2544.

FERREIRA, S. M. & VAN AARDE, R. J. 2008. A rapid method to estimate population variables for african elephants. *Journal of Wildlife Management,* 72**,** 822-829.

FINCH, C. E. 1990. *Longevity, senescence, and the genome,* Chicago, University of Chicago.

GOMPERTZ, B. 1825. On the Nature of the Function expressive of the law of human mortality, and on a new mode of determining the value of life contingencies. *Philosophical Transactions of the Royal Society of London A Mathematical and Physical Sciences,* 115**,** 513–585.

GREEN, R. E. 2004. A new method for estimating the adult survival rate of the Corncrake Crex crex and comparison with estimates from ring-recovery and ring-recapture data. *Ibis,* 146**,** 501-508.

HELIGMAN, L. & POLLARD, J. H. 1980. The age pattern of mortality. *Journal of the Institute of Actuaries,* 107**,** 49-80.

MAKEHAM, W. M. 1860. On the law of mortality and the construction of annuity tables. *Journal of the Institute of Actuaries,* 8**,** 301–310.

RICKER, W. E. 1975. Computation and interpretation of biological statistics of populations. *Fisheries Research Board of Canada,* Bulletin 191**,** 382.

RICKLEFS, R. E. 1997. Comparative demography of new world populations of thrushes (*Turdus spp.*). *Ecological Monographs,* 67**,** 23-43.

SIBLY, R. M., COLLETT, D., PROMISLOW, D. E. L., PEACOCK, D. J. & HARVEY, P. H. 1997. Mortality rates of mammals. *Journal of Zoology,* 243**,** 1-12.

SILER, W. 1979. A competing-risk model for animal mortality. *Ecology,* 60**,** 750-757.

UDEVITZ, M. S. & BALLACHEY, B. E. 1998. Estimating survival rates with age-structure data. *Journal of Wildlife Management,* 62**,** 779-792.

UDEVITZ, M. S. & GOGAN, P. J. P. 2012. Estimating survival rates with time series of standing age-structure data. *Ecology,* 93**,** 726-32.

WITTEN, M. 1987. Information content of biological survival curves arising in aging experiments: some further thoughts. *In:* WOODHEAD, A. & THOMPSON, K. (eds.) *Evolution of Longevity in Animals.* Springer US.
