# Supplementary material for: The adequacy of aging techniques in vertebrates for rapid estimation of population mortality rates from age distributions
Source: Ecol Evol. 2018 Dec 27;9(3):1394–402. doi: 10.1002/ece3.4854 (PMC6374686; doi:10.1002/ece3.4854)
Supplement: Supplementary file 2 [file ECE3-9-1394-s002.docx]

**Appendix S2. Detailed model descriptions and discussion on technical claims**

## A model for the relationship between birth, survival, and age distributions

Consider a well-defined population for which one wants to evaluate its dynamics and potentially simulate or predict its future development. This development is necessarily stochastic and is determined by births within the population and survival of its members. With $\tau$ the maximum age a member of the population can possibly attain, we model a birth within the time interval from time $-\tau$ till time 0 by the probability density function $f_{T}(t) ,$ which takes on the value zero outside this interval. This means that the random time $T$ at which an individual is born within this interval satisfies

$P(T\leq t)=\int_{-\infty}^{t} f_{T}(u)du, -\tau\leq t\leq0, P(T\leq0)=\int_{-\infty}^{\infty} f_{T}(u)du=1.$ eqn1

The density function $f_{T}(t)$ will have a decreasing trend on $[-\tau,0]$ for a population in decline and an increasing one for a growing population.

Furthermore, we model the random survival time $S$ of a newborn via its survival function

$1-F_{S}(s)=P(S>s)=\int_{s}^{\tau} f_{S}(u)du=\int_{s}^{\infty} f_{S}(u)du,$ eqn 2

where $F_{S}(s)$ is the distribution function and $f_{S}(s)$ the density function of $S.$ The survival function $1-F_{S}(s)$ and the birth time density function $f_{T}(t)$ determine the density function $f_{Y}(y)$ of the age $Y$ of an individual chosen at random from the population at time 0, namely by the formula

$f_{Y}(y)=\frac{(1-F_{S}(y))f_{T}(-y)}{\int_{0}^{\infty} (1-F_{S}(t))f_{T}(-t)dt}, -\infty<y<\infty,$ eqn 3

or equivalently

$1-F_{S}(s)=\frac{f_{T}\left( 0 \right)f_{Y}\left( s \right)}{f_{T}\left( -s \right)f_{Y}\left( 0 \right)}, -\infty< s<\infty.$ eqn 4

These equations 3 and 4 describe the relationship between time of birth, survival time, and age, represented by the functions $f_{T}(t), 1-F_{S}(s),$ and $f_{Y}(y),$ respectively. Given two of them, the third one is determined.

The argument leading to equations 3 and 4 now follows.

As $Y$ is the age of an individual chosen at random from the population at time 0, and $\tau$ is the maximum possible age an individual from the population can attain, only newborns from the time interval $[-\tau,0]$ can possibly be observed at time 0. For an individual born in the interval $[-\tau,0]$ to be observed at time 0, this individual has to survive until at least time 0. Only survivors until time 0 can possibly be observed at time 0. Thus, the probability that the age of an individual chosen at random from the population at time 0 equals at most $y,$ is the conditional probability that this individual is born within the interval $[-y,0]$ given the individual survives at least until time 0. In terms of the random variables $S,$ the survival time, and $T,$ the time of birth, of an arbitrary individual, this is the conditional probability that $T$ occurs within the interval $[-y,0]$ given $S$ equals at least $-T.$ In formula

$P(Y\leq y)=P\left( -y\leq T\leq0|S\geq-T \right)$

$=\frac{P\left( S\geq-T, -y\leq T \right)}{P\left( S\geq-T \right)}=\frac{E\left( P\left( S\geq-T, -y\leq T|T \right) \right)}{E\left( P\left( S\geq-T|T \right) \right)}$

$=\frac{E\left( (1-F_{S}(-T))\mathbf{1}_{[-y\leq T]} \right)}{E\left( 1-F_{S}(-T) \right)}=\frac{\int_{-y}^{0} (1-F_{S}(-t))f_{T}(t)dt}{\int_{-\tau}^{0} (1-F_{S}(-t))f_{T}(t)dt},$

where $\mathbf{1}_{[-y\leq T]}$ equals 1 if $-y\leq T$ holds and equals 0 otherwise. As the probability density function $f_{T}(t)$ puts all its mass within $[-\tau,0],$ the function $f_{T}(-t)$ takes on the value zero outside the interval $[0,\tau],$ and we may also write

$P(Y\leq y)=\frac{\int_{0}^{y} (1-F_{S}(t))f_{T}(-t)dt}{\int_{0}^{\infty} (1-F_{S}(t))f_{T}(-t)dt}, y\geq0,$ eqn 5

which by differentiation with respect to $y$ yields equation 3 at those values of $y$ at which $(1-F_{S}(y))f_{T}(-y)$ is continuous. Assuming that $f_{T}(0)$ is positive and $f_{T}(t)$ is continuous at 0 we may rewrite equation 3 as equation 4. By taking $f_{T}(t)=\tau^{-1}\mathbf{1}_{[-\tau\leq t\leq0]},$ the density of the uniform distribution on $[-\tau,0],$ we obtain

$f_{Y}(y)=\frac{1-F_{S}(y)}{\int_{0}^{\infty} (1-F_{S}(t))dt}, y\geq0,$ eqn 6

and

$1-F_{S}(s)=\frac{f_{Y}(s)}{f_{Y}(0)}, s\geq0.$ eqn 7

from equations 3 and 4, respectively. Note that equations 6 and 7 also hold in the limit for $\tau$ tending to infinity, and that they hold for the situation with both birth and survival process stable. These equations 6 and 7 are well-known and are presented e.g. as formulae (1.5) and (1.6) in Van Es et al. (2000). The proof of equations 3 and 4 given above is a slight generalization of the proof of (1.5) and (1.6) from Van Es et al. (2000) given in their Appendix A.1.

Equations 3 and 4 describe how time of birth $T,$ survival time $S,$ and age $Y$ at time 0 influence each other. These three random variables are represented by the probability density function $f_{T}(t)$ of $T,$ the survival function $1-F_{S}(s)$ of $S$, and the probability density function $f_{Y}(y)$ of $Y$. As mentioned earlier, given two of these functions, the third one is determined by the equations 3 and 4. In other words, if $f_{T}(t)$ and $1-F_{S}(s)$ are known, $f_{Y}(y)$ can be computed via equation 3 ; if $1-F_{S}(s)$ and $f_{Y}(y)$ are given, $f_{T}(t)$ can be determined via equation 4; and if $f_{T}(t)$ and $f_{Y}(y)$ are known, $1-F_{S}(s)$ can be computed via equation 4 . Now, in the majority of cases $f_{T}(t)$ can be assessed in some way or reasonably assumed. Consequently, an estimate of the survival function $1-F_{S}(s),$ which is of key interest, can be computed via equations 4, provided $f_{Y}(y)$ can be estimated. To obtain an estimate of $f_{Y}(y)$ one has to measure the age of individuals from a population directly. However, this is almost always impossible and one consequently has to rely on measuring other variables that (hopefully) correlate strongly with age. A general model for this will be discussed in the next section.

## Age and age proxies

As measurement of the age of individuals from a population is almost always impossible, one has to rely on measuring variables $X^{(1)},\ldots,X^{(d)}$ that correlate strongly to age $Y.$ We will call such variables age proxies. Measuring the column vector $X=(X^{(1)},\ldots,X^{(d)})^{T}$ instead of $Y$ introduces extra randomness and hence more uncertainty in the estimate of $f_{Y}(y).$ This influences the accuracy of the prediction of the future development of the population negatively. To what extent this is the case, is the main topic of the present paper, at least for the situation of one proxy ($d=1$). To describe the relationship between age $Y$ and the vector $X$ of age proxies, we first consider the case $d=1$ with $X=X^{(1)}$ a one-dimensional age proxy. For $d=1$ we assume that there exists a strictly monotone, known function $g(y),$ a known positive constant $\sigma,$ and a random variable $\varepsilon$ with known density $f_{\varepsilon}(z),$ distribution function $F_{\varepsilon}(z),$ mean $E\varepsilon=0,$ and variance $E\varepsilon^{2}=1,$ such that

$X=g(Y)+\sigma\varepsilon$ eqn 8

holds with $Y$ and $\varepsilon$ independent. By the rule that the expectation of a conditional probability is the probability itself, this assumption implies

$F_{X}\left( x \right)=P\left( X\leq x \right)=E\left( P\left( X\leq x|Y \right) \right)=E\left( P\left( \frac{X-g(Y)}{\sigma}\leq\frac{x-g(Y)}{\sigma}|Y \right) \right)$

$=E\left( P\left( \varepsilon\leq\frac{x-g(Y)}{\sigma}|Y \right) \right)=E\left( F_{\varepsilon}\left( \frac{x-g(Y)}{\sigma} \right) \right)$ eqn 9

$=\int_{0}^{\infty} F_{\varepsilon}\left( \frac{x-g(y)}{\sigma} \right)f_{Y}(y)dy,$

which by differentiation yields

$f_{X}(x)=\int_{0}^{\infty} \frac{1}{\sigma}f_{\varepsilon}\left( \frac{x-g(y)}{\sigma} \right)f_{Y}(y)dy.$ eqn 10

Equations 9 and 10 are well-known convolution formulae.

For the multidimensional case with $d>1$ we assume that there exist strictly monotone, known functions $g_{1}(y),\ldots,g_{d}(y),$ which we group in the column vector $g(y)=(g_{1}(y),\ldots,g_{d}(y))^{T},$ a known $d\times d$ positive definite matrix $M$ and a $d$-dimensional random vector $\varepsilon$ with known density $f_{\varepsilon}(z),$ mean vector $E\varepsilon=0,$ and covariance matrix $E(\varepsilon\varepsilon^{T})$ equal to the $d\times d$ identity matrix, such that

$X=g(Y)+M\varepsilon$ eqn 11

holds with $Y$ and $\varepsilon$ independent. The convolution equation 10 is generalized in this $d$-dimensional case to

$f_{X}(x)=\int_{0}^{\infty} \frac{1}{|M|}f_{\varepsilon}\left( M^{-1}(x-g(y)) \right)f_{Y}(y)dy,$ eqn 12

where $|M|$ denotes the positive determinant of the matrix $M.$

## The general model

As argued in the second last paragraph of Section 1 of this Appendix, we would like to have an estimate of the density function $f_{Y}(y)$ of the age $Y$ at time 0 in order to compute an estimate of the survival function $1-F_{S}(s)$ of $S$ with the help of equation 4. To be able to apply this formula we also need the density function $f_{T}(t)$ of the time of birth *T* of an individual in the period of length $\tau$ preceding time 0. As mentioned before, in the majority of cases the density function $f_{T}(t)$ can be assessed in some way or reasonably assumed. To obtain an estimate of the density function $f_{Y}(y)$ we need observations and hence we take a random sample of size $n$ from the population under study at time 0. Typically, measuring the age of the individuals in the sample is impossible and we have to rely on measuring age proxies. A model for the relationship between a vector of age proxies and age itself has been presented in Section 2 of this Appendix.

Therefore, we assume that we have obtained $n$ values of the age proxy vector. We view these values as the realizations of $n$ independent and identically distributed copies $X_{1},\ldots,X_{n}$ of the random vector $X.$ Based on this sample and on information about the births in the period $[-\tau,0]$ we would like to estimate the density function $f_{Y}(y)$ of the age $Y$ for all $y\geq0$ and the distribution function $F_{S}(s)$ of the survival time $S$ for all $s\geq0,$ or equivalently the survival function $1-F_{S}(s).$

If we do not make any assumptions at all on the class of survival functions, we have a so-called nonparametric estimation problem at hand. Based on our sample we can estimate the density function $f_{X}(x)$. Estimating the density function $f_{Y}(y)$ using equation 12 is a so-called deconvolution problem. Such problems are notoriously hard in the sense that convergence rates in $n$ are extremely slow, like $\sqrt{\ln n}$; see Carroll and Hall (1988). Nevertheless, once $f_{Y}(y)$ has been estimated, we may use equation 4 to obtain an estimate of the survival function $1-F_{S}(s).$

If restriction of the survival function to a parametric class of survival functions is justified, estimation of the survival function at a $\sqrt{n}$ rate becomes feasible. Let this parametric class of survival functions be parametrized by the vector $\theta$, which takes its values in the parameter set $\Theta$, a subset of $\mathbb{R}^{k}.$ In order to indicate the dependence of the survival time $S$ on $\theta$, we will denote its distribution function and density by $F_{S,\theta}(s)$ and $f_{S,\theta}(s),$ respectively. Accordingly, we denote the densities of $X$ and $Y$ by $f_{X,\theta}(x)$ and $f_{Y,\theta}(y),$ respectively. As in equations 3 and 12 we have

$f_{Y,\theta}(y)=\frac{(1-F_{S,\theta}(y))f_{T}(-y)}{\int_{0}^{\infty} (1-F_{S,\theta}(t))bf_{T}(-t)dt}, y\geq0,$ eqn 13

$f_{X,\theta}(x)=\int_{0}^{\infty} \frac{1}{|M|}f_{\varepsilon}\left( M^{-1}(x-g(y)) \right)f_{Y,\theta}(y)dy, x\in\mathbb{R}^{d}.$ eqn 14

To estimate the distribution function of the age $Y$ and the survival function of the survival time $S$ it now suffices to estimate $\theta.$

A maximizer of $\sum_{i=1}^{n} \ln\left( f_{X,\theta}(X_{i}) \right)$ is called a maximum likelihood estimator of $\theta$ and it is asymptotically efficient, provided $f_{X,\theta}(x)$ satisfies regularity conditions; see e.g. Section 6.2 of Lehmann (1999). Loosely speaking this means that a maximum likelihood estimator is the best possible estimator. More precisely, let $\theta_{0}$ be the "true value" of the parameter and let $\hat{\theta}_{n}=\hat{\theta}_{n}(X_{1},\ldots,X_{n})$ denote a maximizer of $\sum_{i=1}^{n} \ln\left( f_{X,\theta}(X_{i}) \right)$ that converges to $\theta_{0}$ as the sample size $n$ tends to infinity and the observations $X_{1},\ldots,X_{n}$ have density $f_{X,\theta_{0}}(x).$ Then the distribution of $\sqrt{n}(\hat{\theta}_{n}-\theta_{0})$ converges (as the sample size $n$ tends to infinity and the observations $X_{1},\ldots,X_{n}$ have density $f_{X,\theta_{0}}(x)$) to a $k$-dimensional normal distribution with mean vector 0 and covariance matrix $I^{-1}(\theta_{0}),$ with $I(\theta_{0})$ the Fisher information matrix; see e.g. section 2.7 of Lehmann (1999). Here the covariance matrix $I^{-1}(\theta_{0})$ is minimal and $\sqrt{n}$ is the fastest possible rate.

We summarize the assumptions for the general model with a parametric class of survival functions and without covariates as follows.

**General parametric model**

- With $\tau$ the maximum possible age, the random time $T$ of a birth within the interval $[-\tau,0]$ has known probability density $f_{T}(t).$
- The survival time $S$ of a (randomly chosen) newborn has survival function $1-F_{S,\theta}(s)$ with unknown $\theta\in\Theta\subset\mathbb{R}^{k}.$
- The vector $X$ of $d$ age proxies and age $Y$ of a randomly chosen individual at time 0 are related by equation 11 with $g(y)$ having known monotone functions as its $d$ components.
- The regression error $\varepsilon$ has mean vector 0, covariance matrix the identity matrix, and known density $f_{\varepsilon}(z).$
- The matrix $M$ is known and positive definite.
- The observations $x_{1},\ldots,x_{n}$ are viewed as realizations of the independent identically distributed random vectors $X_{1},\ldots,X_{n},$ which have density $f_{X,\theta}(x)$ as given in equation 14.

## 4 Specific model with constant mortality rate

Assume an exponential distribution is an appropriate model for the survival time $S$ of a newborn (chosen at random) from the population we consider. This means that we assume the existence of a positive number $\lambda$ with

$1-F_{S,\lambda}(s)=P(S>s)=e^{-\lambda s}=(1-m)^{s}, s\geq0,$ eqn 15

and $\lambda=-ln(1-m),$ which implies that the density of $S$ satisfies

$f_{S,\lambda}(s)=\lambda e^{-\lambda s}, s\geq0.$ eqn 16

Given a newborn with survival time $S$ has lived up to and including time $s,$ the conditional probability this individual will die at or before time $s+1$ equals

$P\left( S\leq s+1|S>s \right)=\frac{P(s<S\leq s+1)}{P(s<S)}$ eqn 17

$=\frac{F_{S,\lambda}(s+1)-F_{S,\lambda}(s)}{1-F_{S,\lambda}(s)}=1-e^{-\lambda}=m.$

Loosely speaking $m$ is the probability that an individual dies within the next time unit. Therefore $m$ is called the mortality rate and $0<m<1$ holds. Note that this mortality rate is constant with age.

Although this simplification is not strictly necessary from a mathematical perspective (i.e. models allowing for much more general or complex relationships between survival and age, and thus mortality rate and age, are feasible as outlined in Section 3 above), we apply it here, as it dramatically reduces the required sample size (see also Section 3). In many cases this is also a reasonable assumption for two reasons. Firstly, some species do show either a constant mortality rate or a roughly constant mortality rate before reaching a very advanced age (6 out of 23 vertebrates investigated in (Jones et al., 2014)). Secondly, although in the remaining species the relationship between age and mortality rate varies, it often increases exponentially with age, resulting in few individuals within wildlife populations living long enough to be impacted by a substantially increased mortality rate with age. Hence, estimating age-specific mortality rates often require large, long-term data sets, be it mark-recapture data or population age distribution data. Such data are, however, available for very few species only (see (Jones et al., 2014)). Most case studies lack sufficient data and thus estimate mortality rate based on the assumption of a constant adult mortality. Given the relatively low number of individuals affected by advanced-age-related changes in mortality the potential impact on the population dynamics of such assumption may also be limited. Finally, this simplification is notably warranted in the present case, where the aim of the exercise is primarily focused on evaluating the suitability of a range of popular or potentially promising vertebrate aging techniques to establish population age distributions with the purpose of estimating survival functions.

To simplify further we assume that the population is completely stable, in the sense that $f_{T}(t)$ is the uniform density on $[-\tau,0]$ with $\tau$ tending to infinity. In view of equations 6 and 13 we have

$f_{Y,\lambda}(y)=\frac{1-F_{S,\lambda}(y)}{\int_{0}^{\infty} (1-F_{S,\lambda}(t))dt}=\frac{e^{-\lambda y}}{1/\lambda}=\lambda e^{-\lambda y}, y\geq0.$ eqn 18

This means that the age $Y$ of an arbitrary individual from the population has the same exponential distribution as the survival time $S$ of a newborn.

We have specialized the general model from the preceding section by taking $Y$ and $S$ exponentially distributed, both with parameter $\lambda.$ We specialize the general model further to the specific model used in the main text by assuming $d=1,$ i.e., just one age proxy is measured. Furthermore, we assume that the standardized error $\varepsilon$ in the regression equation 8 has standard normal density $\varphi(z)$ and that $g(y)$ is linear, i.e., that there exist known constants $\alpha$ and $\beta$ with

$g(y)=\alpha+\beta y , y>0.$ eqn 19

So, we make the classic assumption that the dependence of the age proxy $X$ on the age $Y$ is described via linear regression with normal error. Note that only for $\beta\neq0$ the distribution of the age proxy $X$ depends on the distribution of the age $Y$ and hence on $\lambda.$ Substitution in equation 14 shows that the density of the age proxy $X$ becomes

$f_{X,\lambda}(x)=\int_{0}^{\infty} \frac{1}{\sigma}\varphi\left( \frac{x-\alpha-\beta y}{\sigma} \right)\lambda e^{-\lambda y}dy, x\mathbb{\in R.}$ eqn 20

We can now summarize the assumptions for the specific parametric model used in the main text as:

**Specific parametric model**

- The random time $T$ of a birth has a uniform distribution on the interval $[-\tau,0]$ with $\tau$ tending to infinity.
- The survival time $S$ of a (randomly chosen) newborn has survival function $1-F_{S,\lambda}(s)=e^{-\lambda s}$ with unknown positive parameter $\lambda.$
- Age proxy $X$ and age $Y$ of a randomly chosen individual are related by

$X=\alpha+\beta Y+\sigma\varepsilon$ eqn 21

with known regression parameters $\alpha$ and $\beta\neq0.$

- The regression error $\varepsilon$ has standard normal density $\varphi(z).$
- The standard deviation $\sigma$ is known and positive.
- The observations $x_{1},\ldots,x_{n}$ are viewed as realizations of the independent identically distributed random variables $X_{1},\ldots,X_{n},$ which have density $f_{X,\lambda}(x)$ as given in equation 20.

## 5 Estimation of mortality rate

Writing $\mu=\sigma\lambda/|\beta|$ we note that estimation of the mortality rate

$m=1-exp(-\lambda)=1-exp(-|\beta|\mu/\sigma)$ eqn 22

is equivalent to estimation of the parameter $\mu.$ The correlation between age proxy $X$ and age $Y$ equals 1/$\sqrt{1+ \mu^{2}}$, and hence this correlation is a monotone function of $\mu.$ The smaller$\mu$ the better the age proxy. Therefore, we propose to call $\mu$ the proxy coefficient. In this Section we construct an optimal estimator for$\mu$ and we show how this estimator can be converted into an optimal estimator for $m$*.* First we compute the so-called Fisher information for $\mu$*.*

With $\Phi(z)$ denoting the standard normal distribution function, equation 20 can be rewritten in terms of the proxy coefficient $\mu$ as

$f_{X,\lambda}(x)=\int_{0}^{\infty} \frac{\lambda}{\sigma\sqrt{2\pi}}\exp\left( -\frac{1}{2}\left( \frac{x-\alpha-\beta y}{\sigma} \right)^{2}-\lambda y \right)dy$

$=\int_{0}^{\infty} \frac{\lambda}{\sigma\sqrt{2\pi}}\exp\left( -\frac{1}{2}\left( \frac{\beta}{\sigma}y+\frac{\sigma\lambda}{\beta}-\frac{x-\alpha}{\sigma} \right)^{2}+\frac{1}{2}\left( \frac{\sigma\lambda}{\beta} \right)^{2}-\frac{\lambda}{\beta}(x-\alpha) \right)dy$

$=\frac{\lambda}{\sigma}\exp\left( \frac{1}{2}\left( \frac{\sigma\lambda}{\beta} \right)^{2}-\frac{\lambda}{\beta}(x-\alpha) \right)$ eqn 23

$\times\int_{0}^{\infty} \frac{1}{\sqrt{2\pi}}\exp\left( -\frac{1}{2}\left( -\frac{|\beta|}{\sigma}y-\frac{\sigma\lambda}{|\beta|}+\frac{\beta}{|\beta|}\frac{x-\alpha}{\sigma} \right)^{2} \right)dy$

$=\frac{\lambda}{|\beta|}\exp\left( \frac{1}{2}\left( \frac{\sigma\lambda}{\beta} \right)^{2}-\frac{\lambda}{\beta}(x-\alpha) \right)$

$\times\int_{-\infty}^{0} \frac{1}{\sqrt{2\pi}}\exp\left( -\frac{1}{2}\left( z-\frac{\sigma\lambda}{|\beta|}+\frac{\beta}{|\beta|}\frac{x-\alpha}{\sigma} \right)^{2} \right)dz$

$=\frac{\lambda}{|\beta|}\exp\left( \frac{1}{2}\left( \frac{\sigma\lambda}{\beta} \right)^{2}-\frac{\lambda}{\beta}(x-\alpha) \right)\Phi\left( \frac{\beta}{|\beta|}\frac{x-\alpha}{\sigma}-\frac{\sigma\lambda}{|\beta|} \right)$

$=\frac{\mu}{\sigma}\exp\left( \frac{1}{2}\mu^{2}-\mu\frac{\beta}{|\beta|}\frac{x-\alpha}{\sigma} \right)\Phi\left( \frac{\beta}{|\beta|}\frac{x-\alpha}{\sigma}-\mu\right)=f_{\mu}\left( x \right).$

The logarithmic derivative of this density with respect to *µ* equals

$\frac{\partial}{\partial\mu}\ln\left( f_{\mu}(x) \right)$

$=\frac{\partial}{\partial\mu}\left\{ \ln\mu+\frac{1}{2}\mu^{2}-\mu\frac{\beta}{|\beta|} \frac{x-\alpha}{\sigma}+ln\Phi\left( \frac{\beta}{|\beta|} \frac{x-\alpha}{\sigma}-\mu\right) \right\}$

$=\frac{1}{\mu}+\mu-\frac{\beta}{|\beta|} \frac{x-\alpha}{\sigma}-\frac{\varphi}{\Phi}\left( \frac{\beta}{|\beta|} \frac{x-\alpha}{\sigma}-\mu\right)$ eqn 24

$=\frac{1}{\mu}-\psi\left( \frac{\beta}{|\beta|} \frac{x-\alpha}{\sigma}-\mu\right)$

with $\psi(z) = z +\varphi/\Phi\left( z \right)$ and with $\varphi/\Phi$(*z*) written as short hand for $\varphi(z)/\Phi\left( z \right)$.

By definition the Fisher information $J(\mu)$ for $\mu$ is the second moment under $\mu$ of the logarithmic derivative of the density of $X$ at $x=X$ with respect to $\mu$*.* In view of equation 24 this means

$J(\mu)=E_{\mu}\left( \left( \frac{\partial}{\partial\mu}\ln f_{\mu}(X) \right)^{2} \right)$

$=E_{\mu}\left( \left( \frac{1}{\mu}-\psi\left( \frac{\beta}{|\beta|} \frac{X-\alpha}{\sigma}-\mu\right) \right)^{2} \right)$

$=\int_{-\infty}^{\infty} \left( \frac{1}{\mu}-\psi\left( \frac{\beta}{|\beta|} \frac{x-\alpha}{\sigma}-\mu\right) \right)^{2}f_{\mu}(x)dx$ eqn 25

$=\int_{-\infty}^{\infty} \left( \frac{1}{\mu^{2}}-\frac{2}{\mu}\psi\left( \frac{\beta}{|\beta|} \frac{x-\alpha}{\sigma}-\mu\right)+\left[ \psi\left( \frac{\beta}{|\beta|} \frac{x-\alpha}{\sigma}-\mu\right) \right]^{2} \right)f_{\mu}(x)dx$

$=\frac{1}{\mu^{2}}-\frac{2}{\mu}\int_{-\infty}^{\infty} \psi\left( \frac{\beta}{|\beta|} \frac{x-\alpha}{\sigma}-\mu\right)f_{\mu}(x)dx$

$+\int_{-\infty}^{\infty} \left[ \psi\left( \frac{\beta}{|\beta|} \frac{x-\alpha}{\sigma}-\mu\right) \right]^{2}f_{\mu}(x)dx.$

Furthermore, we have

$\int_{-\infty}^{\infty} \psi\left( \frac{\beta}{|\beta|} \frac{x-\alpha}{\sigma}-\mu\right)f_{\mu}(x)dx$

$=\int_{-\infty}^{\infty} \left\{ \frac{\beta}{|\beta|} \frac{x-\alpha}{\sigma}-\mu+\frac{\varphi}{\Phi}\left( \frac{\beta}{|\beta|} \frac{x-\alpha}{\sigma}-\mu\right) \right\}f_{\mu}(x)dx$

$=E_{\mu}\left( \frac{\beta}{|\beta|} \frac{X-\alpha}{\sigma}-\mu\right)$

$+\int_{-\infty}^{\infty} \frac{\varphi}{\Phi}\left( \frac{\beta}{|\beta|} \frac{x-\alpha}{\sigma}-\mu\right)\frac{\mu}{\sigma}\exp\left( \frac{1}{2}\mu^{2}-\mu\frac{\beta}{|\beta|} \frac{x-\alpha}{\sigma} \right)$

$\times\Phi\left( \frac{\beta}{|\beta|} \frac{x-\alpha}{\sigma}-\mu\right)dx$

$=E_{\mu}\left( \frac{\beta}{|\beta|} \frac{\beta Y+\sigma\varepsilon}{\sigma}-\mu\right)$

$+\int_{-\infty}^{\infty} \varphi\left( \frac{\beta}{|\beta|} \frac{x-\alpha}{\sigma}-\mu\right)\frac{\mu}{\sigma}\exp\left( \frac{1}{2}\mu^{2}-\mu\frac{\beta}{|\beta|} \frac{x-\alpha}{\sigma} \right)dx$

$=\frac{|\beta|}{\sigma\lambda}-\mu+\int_{-\infty}^{\infty} \varphi(y) \frac{\mu}{\sigma}\exp\left( \frac{1}{2}\mu^{2}-\mu(y+\mu) \right)\sigma dy$

$=\frac{1}{\mu}-\mu+\mu\int_{-\infty}^{\infty} \frac{1}{\sqrt{2\pi}}\exp\left( -\frac{1}{2}y^{2}-\frac{1}{2}\mu^{2}-\mu y \right)dy$

$=\frac{1}{\mu}-\mu+\mu\int_{-\infty}^{\infty} \varphi(y+\mu)dy=\frac{1}{\mu}$ eqn 26

and

$\int_{-\infty}^{\infty} \left[ \psi\left( \frac{\beta}{|\beta|} \frac{x-\alpha}{\sigma}-\mu\right) \right]^{2}f_{\mu}(x)dx$

$=\int_{-\infty}^{\infty} \left\{ \frac{\beta}{|\beta|} \frac{x-\alpha}{\sigma}-\mu+\frac{\varphi}{\Phi}\left( \frac{\beta}{|\beta|} \frac{x-\alpha}{\sigma}-\mu\right) \right\}^{2}$

$\times\frac{\mu}{\sigma}\exp\left( \frac{1}{2}\mu^{2}-\mu\frac{\beta}{|\beta|} \frac{x-\alpha}{\sigma} \right)\Phi\left( \frac{\beta}{|\beta|} \frac{x-\alpha}{\sigma}-\mu\right)dx$

$=\int_{-\infty}^{\infty} \left\{ y+\frac{\varphi}{\Phi}\left( y \right) \right\}^{2}\frac{\mu}{\sigma}\exp\left( \frac{1}{2}\mu^{2}-\mu(y+\mu) \right)\Phi\left( y \right)\sigma dy$ eqn 27

$=\mu e^{-\frac{1}{2}\mu^{2}}\int_{-\infty}^{\infty} \left\{ y^{2}+2y\frac{\varphi}{\Phi}\left( y \right)+\left[ \frac{\varphi}{\Phi}\left( y \right) \right]^{2} \right\}e^{-\mu y}\Phi\left( y \right)dy.$

By partial integration and in view of $E\left( \varepsilon^{2} \right)=1$, $E\varepsilon=0$, we obtain

$\int_{-\infty}^{\infty} y^{2}e^{-\mu y}\Phi\left( y \right)dy$

$=\left[ \left( -\frac{y^{2}}{\mu}-\frac{2y}{\mu^{2}}-\frac{2}{\mu^{3}} \right)e^{-\mu y}\Phi\left( y \right) \right]_{-\infty}^{\infty}$

$-\int_{-\infty}^{\infty} \left( -\frac{y^{2}}{\mu}-\frac{2y}{\mu^{2}}-\frac{2}{\mu^{3}} \right)e^{-\mu y}\varphi\left( y \right)dy$

$=\frac{1}{\sqrt{2\pi}}\int_{-\infty}^{\infty} \left( \frac{y^{2}}{\mu}+\frac{2y}{\mu^{2}}+\frac{2}{\mu^{3}} \right)e^{-\mu y-\frac{1}{2}y^{2}}dy$ eqn 28

$=\frac{1}{\sqrt{2\pi}}\int_{-\infty}^{\infty} \left( \frac{y^{2}}{\mu}+\frac{2y}{\mu^{2}}+\frac{2}{\mu^{3}} \right)e^{-\frac{1}{2}(y+\mu)^{2}}dy e^{\frac{1}{2}\mu^{2}}$

$=\frac{1}{\sqrt{2\pi}}\int_{-\infty}^{\infty} \left( \frac{(z-\mu)^{2}}{\mu}+\frac{2(z-\mu)}{\mu^{2}}+\frac{2}{\mu^{3}} \right)e^{-\frac{1}{2}z^{2}}dz e^{\frac{1}{2}\mu^{2}}$

$=\left( \frac{1+\mu^{2}}{\mu}+\frac{-2\mu}{\mu^{2}}+\frac{2}{\mu^{3}} \right)e^{\frac{1}{2}\mu^{2}}=\left( \mu-\frac{1}{\mu}+\frac{2}{\mu^{3}} \right)e^{\frac{1}{2}\mu^{2}}.$

In a similar way we obtain

$\int_{-\infty}^{\infty} 2y\frac{\varphi}{\Phi}\left( y \right)e^{-\mu y}\Phi\left( y \right)dy=\frac{1}{\sqrt{2\pi}}\int_{-\infty}^{\infty} 2y e^{-\mu y-\frac{1}{2}y^{2}}dy$

$=\frac{1}{\sqrt{2\pi}}\int_{-\infty}^{\infty} 2y e^{-\frac{1}{2}(y+\mu)^{2}}dy e^{\frac{1}{2}\mu^{2}}$ eqn 29

$=\frac{1}{\sqrt{2\pi}}\int_{-\infty}^{\infty} 2(z-\mu)e^{-\frac{1}{2}z^{2}}dz e^{\frac{1}{2}\mu^{2}}=-2\mu e^{\frac{1}{2}\mu^{2}}$

and

$\int_{-\infty}^{\infty} \left[ \frac{\varphi}{\Phi}\left( y \right) \right]^{2}e^{-\mu y}\Phi\left( y \right)dy=\frac{1}{\sqrt{2\pi}}\int_{-\infty}^{\infty} \frac{\varphi}{\Phi}(y) e^{-\mu y-\frac{1}{2}y^{2}}dy$ eqn 30

$=\frac{1}{\sqrt{2\pi}}\int_{-\infty}^{\infty} \frac{\varphi}{\Phi}(y) e^{-\frac{1}{2}(y+\mu)^{2}}dy e^{\frac{1}{2}\mu^{2}}=\int_{-\infty}^{\infty} \frac{\varphi}{\Phi}(y)\varphi(y+\mu)dy e^{\frac{1}{2}\mu^{2}}.$

Adding up equations 28, 29 and 30, and combining the result with equation 27 we get

$\int_{-\infty}^{\infty} \left[ \psi\left( \frac{\beta}{|\beta|} \frac{x-\alpha}{\sigma}-\mu\right) \right]^{2}f_{\mu}(x)dx$

$=\mu\left\{ \mu-\frac{1}{\mu}+\frac{2}{\mu^{3}}-2\mu+\int_{-\infty}^{\infty} \frac{\varphi}{\Phi}(y)\varphi(y+\mu)dy \right\}$ eqn 31

$=\frac{2}{\mu^{2}}-1-\mu^{2}+\mu\int_{-\infty}^{\infty} \frac{\varphi}{\Phi}(y)\varphi(y+\mu)dy.$

Combining equations 25, 26 and 31 we see that the Fisher information for the proxy coefficient $\mu$ equals

$J(\mu)=\frac{1}{\mu^{2}}-2\frac{1}{\mu} \frac{1}{\mu}+\frac{2}{\mu^{2}}-1-\mu^{2}+\mu\int_{-\infty}^{\infty} \frac{\varphi}{\Phi}(y)\varphi(y+\mu)dy$

$=\frac{1}{\mu^{2}}-1-\mu^{2}+\mu\int_{-\infty}^{\infty} \frac{\varphi}{\Phi}\left( y \right)\varphi\left( y+\mu\right)dy.$ eqn 32

By equation 24 we see that ${\partial ln(f}_{\mu}(X))/\partial\mu$ is unequal to 0 with probability 1. Consequently, the Fisher information $J(\mu)$, defined in equation 25, is positive, as it is the second moment of the random variable ${\partial ln(f}_{\mu}(X))/\partial\mu$*.* Furthermore, $J(\mu)$ is finite, as the integral in equation 32 is, which is shown by the lemma in Section 6 below.

Classic asymptotic statistical theory states that, under regularity conditions, the maximum likelihood method yields asymptotically efficient estimators in parametric models, like we have here. An estimator $\hat{\mu}_{n}$ of $\mu$ is asymptotically efficient if for large values of the sample size $n$ the normed difference $\sqrt{n}\left( \hat{\mu}_{n}-\mu\right)$ between the estimator and the true value of the parameter is approximately normally distributed with mean 0 and minimal variance. This minimal variance equals $1/J(\mu)$ with $J(\mu)$ the Fisher information for the proxy coefficient $\mu$ as computed in equation 32; see Sections 7.1 and 7.4 of Lehmann (1999) for more details.

Equating to 0 the derivative of $\sum_{i=1}^{n} \ln f_{\mu}\left( x_{i} \right)$ with respect to $\mu$ we obtain the maximum likelihood equation for *µ*, namely (cf. equation 24)

$\frac{1}{n}\sum_{i=1}^{n} \frac{d}{d\mu}\ln f_{\mu}\left( x_{i} \right)=\frac{1}{\mu}- \frac{1}{n}\sum_{i=1}^{n} \psi\left( \frac{\beta}{|\beta|} \frac{x_{i}-\alpha}{\sigma}-\mu\right)=0, \mu>0.$ eqn 33

A positive root of it is a maximum likelihood estimate for $\mu$ from which a maximum likelihood estimate for $m$ can be derived via equation 22. Typically, but not always, a maximum likelihood estimator is asymptotically efficient. In the present case we have been able to prove that the maximum likelihood equation has a unique solution for large sample sizes $n$, provided the true value of $\mu$ is less than 2. However, we have not been able to prove this for $\mu$ at least 2. Consequently, we cannot be certain that a solution of the maximum likelihood equation will yield an efficient estimate of $\mu$, as the true value of $\mu$ is likely to be larger than 2 and hence the maximum likelihood equation might have more than one root. Therefore, we have chosen another approach to efficiently estimate $\mu$.

As before, let $X_{1},\ldots,X_{n}$ denote the random variables that have generated our observations $x_{1},\ldots,x_{n}$. We denote the sample mean by

$$\bar{X}_{n}=\frac{1}{n}\sum_{i=1}^{n} X_{i}$$

and introduce a preliminary estimator $\bar{\mu}_{n}$of $\mu$ by

$\bar{\mu}_{n}=\frac{\beta\sigma}{\left| \beta\right|\left( \bar{X}_{n}-\alpha\right)} .$ eqn 34

From equation 21 we obtain $E\left( \overline{X}_{n} \right)= \alpha+ \beta/\lambda$ and hence the central limit theorem yields that $\sqrt{n}\left( \frac{\overline{X}_{n}-\alpha}{\beta}-\frac{1}{\lambda} \right)$ is asymptotically normal with mean 0 and variance $\lambda^{-2}+ \sigma^{2}\beta^{-2}$. By the so called *δ*-method (see Theorem 2.5.2 of Lehmann (1999)) this implies that $\sqrt{n}\left( \overline{\mu}_{n}-\mu\right)$is asymptotically normal with mean 0 and variance $\mu^{2}+\mu^{4}$. Therefore, our preliminary estimator $\overline{\mu}_{n}$ from equation 34 is called a $\sqrt{n}$-consistent estimator of $\mu$. Subsequently, we estimate the Fisher information $J(\bar{\mu}_{n})$ at $\bar{\mu}_{n}$ by (cf. equation 25)

$\hat{J}_{n}= \frac{1}{n}\sum_{i=1}^{n} \left\{ \frac{1}{\bar{\mu}_{n}}- \psi\left( \frac{\beta}{|\beta|} \frac{X_{i}-\alpha}{\sigma}-\bar{\mu}_{n} \right) \right\}^{2}$ eqn 35

and we define our final estimator $\hat{\mu}_{n}$ of $\mu$ as

$\hat{\mu}_{n}=\left( 1+\frac{1}{\hat{J}_{n}} \right)\bar{\mu}_{n}-\frac{1}{n\hat{J}_{n}}\sum_{i=1}^{n} \frac{\varphi}{\Phi}\left( \frac{\beta}{|\beta|}\frac{X_{i}-\alpha}{\sigma}-\bar{\mu}_{n} \right)$. eqn 36

By Proposition 2.1.1 of Bickel et al. (1993) we are dealing here with a regular parametric model. Theorem 2.5.2 and the paragraph following the proof of this Theorem of Bickel et al. (1993) then show that $\hat{\mu}_{n}$from equation 36 is asymptotically efficient. (Note that the discretization step needed to complete the efficiency proof of $\hat{\mu}_{n}$in this theorem is not applied in practice; and we do not do this here either.)

In view of equation 22 this efficient estimator $\hat{\mu}_{n}$of *μ* can be converted into an estimator $\hat{m}_{n}$ for $m$ by

$\hat{m}_{n}=1-exp\left( -\frac{|\beta|\hat{\mu}_{n}}{\sigma} \right).$ eqn 37

By the *δ*-method and Theorem 7.4.1 of Lehmann (1999) this estimator $\hat{m}_{n}$ is asymptotically efficient, which means that $\sqrt{n}\left( \hat{m}_{n}-m \right)$ is approximately normal for large sample sizes with mean 0 and minimal variance $1/I(m)$ with

$I(m)=\frac{\sigma^{2}J\left( -\frac{\sigma}{|\beta|} ln(1-m) \right)}{\beta^{2}(1-m)^{2}}$ eqn 38

the Fisher information for $m$*,* where $J\left( -\frac{\sigma}{|\beta|} ln(1-m) \right)$ is the Fisher information for $\mu$ evaluated at $\mu=-\frac{\sigma}{|\beta|} ln(1-m)$.

By taking many samples of size $n$ from the set of numbers ${\{x}_{1},\ldots,x_{n}\}$, computing the corresponding estimates of $m$, and estimating in this way the distribution of $\hat{m}_{n},$ we may construct a bootstrap confidence interval for $m$.

As an alternative 95%-confidence interval for $m$ we mention

$\left[ \hat{m}_{n}-\frac{1.96}{\sqrt{n I\left( \hat{m}_{n} \right)}} , \hat{m}_{n}+\frac{1.96}{\sqrt{n I\left( \hat{m}_{n} \right)}} \right],$ eqn 39

where the function$I\left( m \right)$ is evaluated at $\hat{m}_{n}.$The asymptotic efficiency of $\hat{m}_{n}$implies $\lim_{n\to\infty}P_{\mu}\left( \sqrt{nI\left( \hat{m}_{n} \right)} \left( \hat{m}_{n}-m \right)\leq z \right)=\Phi\left( z \right), -\infty<z<\infty.$ eqn 40

In particular this means

$\lim_{n\to\infty}P_{\mu}\left( -1.96\leq\sqrt{nI\left( \hat{m}_{n} \right)} \left( \hat{m}_{n}-m \right)\leq1.96 \right)$

$=\Phi(1.96)-\Phi(-1.96)=2\Phi(1.96)-1\approx0.95,$ eqn 41

which proves that the interval from equation 39 is a(n approximate) 95%-confidence interval indeed. The 95%- confidence range (CR) may be calculated by deducting the lower limit from the upper limit and equals

$CR(95)=\frac{3.92}{\sqrt{n I\left( \hat{m}_{n} \right)}}.$ eqn 42

We define the empirical 95% error percentage as

$EEP\left( 95 \right)=\frac{CR\left( 95 \right)}{\hat{m}_{n}}\times100\%= \frac{392}{\sqrt{n}} \times\frac{1}{\hat{m}_{n}\sqrt{I\left( \hat{m}_{n} \right)}} \%,$ eqn 43

which estimates the theoretical 95% error percentage

$EP\left( 95 \right)= \frac{392}{\sqrt{n}} \times\frac{1}{m\sqrt{I\left( m \right)}} \%,$ eqn 44

with$m$ the true value of the mortality rate. We used $EEP(95)$ to assess the accuracy of mortality rate estimation from age distributions with large values indicating high variation and thus lower accuracy.

##

## 6 Numerical Computation Fisher Information

For the construction of Fig. 1 and Table 2 we need to compute $1/(m\sqrt{I(m)})$ with the Fisher information $I(m)$ as defined in equation 38. Numerically this poses some problems. The difficulty is in the computation for *μ* positive of the integral $K(\mu)$ defined by (cf. equation 32)

$K(\mu)=\int_{-\infty}^{\infty} \frac{\varphi(x)}{\Phi(x)}\varphi(x+\mu)dx, J(\mu)=\frac{1}{\mu^{2}}-1-\mu^{2}+\mu K(\mu),$ eqn 45

as computation of the integrand involves division of two extremely small positive numbers for values of *x* that are negative and large in absolute value. The computation of $K(\mu)$ is facilitated by the following result, which we have applied with $c$ = 8 in Table 2 and Fig. 1.

### Lemma

*We define*

$L(\mu)=\mu+\int_{-\mu-c}^{-\mu+c} \left( x+\frac{\varphi(x)}{\Phi(x)} \right)\varphi(x+\mu)dx$ eqn 46

*with* $c$ *a positive constant. Then, for μ with*$0< \mu<c$ *we have*

$L(\mu)<K(\mu)<L(\mu)+\left( 1+\frac{1}{c}+\frac{1}{c^{2}} \right)\varphi(c).$ eqn 47

The difference between the upper- and lower-bound of$K(\mu)$ is below 10^-8^ for all combinations of $m$ and $|\beta/\sigma|$used to construct Fig. 1 (where $K\left( \mu\right)$ itself varies between 0.9 and 8.1 across all these combinations). The boundary is thus considered extremely sharp. Indeed, use of either the upperbound, the lowerbound or their average, produces virtually identical results both for Fig. 1 and Table 2.

### Proof

According to equation 10 of Gordon (Gordon, 1941) the following inequalities hold for *negative* values of $x$,

$0\leq x+\frac{\varphi(x)}{\Phi(x)}\leq-\frac{1}{x}.$ eqn 48

The first inequality clearly holds for positive values of *x* as well. This implies that

$K(\mu)-L(\mu)=\mu+\int_{-\infty}^{\infty} \left( x+\frac{\varphi(x)}{\Phi(x)} \right)\varphi(x+\mu)dx-L(\mu)$ eqn 49

$=\int_{-\infty}^{-\mu-c} \left( x+\frac{\varphi(x)}{\Phi(x)} \right)\varphi(x+\mu)dx+\int_{-\mu+c}^{\infty} \left( x+\frac{\varphi(x)}{\Phi(x)} \right)\varphi(x+\mu)dx$

is positive and hence that the first inequality from equation 47 holds. The second inequality from equation 48 yields

$\int_{-\infty}^{-\mu-c} \left( x+\frac{\varphi(x)}{\Phi(x)} \right)\varphi(x+\mu)dx\leq\int_{-\infty}^{-\mu-c} \left( -\frac{1}{x} \right)\varphi(x+\mu)dx$

$\leq\frac{1}{\mu+c}\Phi(-c)\leq\frac{1}{(\mu+c)c}\varphi(c),$ eqn 50

where the last inequality follows from the first inequality from equation 48. The function $\varphi(x)/\Phi(x)$ is strictly decreasing on the whole real line, as its derivative is negative as may be verified with the help of the first inequality from equation 48. Consequently, for $\mu<c$ we have

$\int_{-\mu+c}^{\infty} \frac{\varphi(x)}{\Phi(x)}\varphi(x+\mu)dx\leq\frac{\varphi(-\mu+c)}{\Phi(-\mu+c)}\left( 1-\Phi(c) \right)$

$<\frac{\varphi(0)}{\Phi(0)}\left( 1-\Phi(c) \right)=\sqrt{\frac{2}{\pi}}\left( 1-\Phi(c) \right)<\frac{1}{c}\varphi(c),$ eqn 51

where, again, the last inequality follows from the first inequality of equation 48. Furthermore, we have

$\int_{-\mu+c}^{\infty} x\varphi(x+\mu)dx=\left[ -\varphi(x+\mu)-\mu\Phi(x+\mu) \right]_{-\mu+c}^{\infty}<\varphi(c).$ eqn 52

Combining equations 48 through 51 we arrive at the second inequality from equation 47.

# References

BICKEL, P. J., KLAASSEN, C. A. J., RITOV, Y. & WELLNER, J. A. 1993. Efficient and adaptive estimation for semiparametric models. *MR1245941.* 1998 ed. New York: Springer, The Johns Hopkins University Press, Baltimore.

CARROLL, R. J. & HALL, P. 1988. Optimal rates of convergence for deconvolving a density. *Journal of the American Statistical Association,* 83**,** 1184-1186.

GORDON, R. D. 1941. Values of Mills' ratio of area to bounding ordinate and of the normal probability integral for large values of the argument. *The Annals of Mathematical Statistics,* 12**,** 364-366.

JONES, O. R., SCHEUERLEIN, A., SALGUERO-GOMEZ, R., CAMARDA, C. G., SCHAIBLE, R., CASPER, B. B., DAHLGREN, J. P., EHRLEN, J., GARCIA, M. B., MENGES, E. S., QUINTANA-ASCENCIO, P. F., CASWELL, H., BAUDISCH, A. & VAUPEL, J. W. 2014. Diversity of ageing across the tree of life. *Nature,* 505**,** 169-173.

LEHMANN, E. L. 1999. *Elements of large-sample theory,* New York, Springer.

VAN ES, B., KLAASSEN, C. A. J. & OUDSHOORN, K. 2000. Survival analysis under cross-sectional sampling: length bias and multiplicative censoring. *Journal of Statistical Planning and Inference,* 91**,** 295-312.
