# Supplementary material for: The adequacy of aging techniques in vertebrates for rapid estimation of population mortality rates from age distributions
Source: Ecol Evol. 2018 Dec 27;9(3):1394–402. doi: 10.1002/ece3.4854 (PMC6374686; doi:10.1002/ece3.4854)
Supplement: Supplementary file 3 [file ECE3-9-1394-s003.docx]

**Appendix S3. Background information on seven different ageing techniques.**

Eight aging techniques were reviewed because they are well known indicators that to some extent correlate with age (i.e. telomere length, otolith ring count, otolithometry, age-length keys and skeletochronology), or are promising indicators in determining age with low errors (i.e. DNA mythelation, sjTREC and racemization).

### Telomere length

Telomeres are highly conservative DNA tandems at the ends of eukaryotic linear chromosomes. Considering the general principle of telomere shortening with aging, telomere length has mainly been employed as a biomarker for biological age in humans and other mammals, birds and fishes and less often in other animal taxa. Its correlation with age was reviewed in Dunshea et al. (2011). Besides age, telomere length also varies with an individual’s health status and can for instance be used as an indicator of oxidative stress (Haussmann et al., 2005). Thus, telomere shortening may provide important information on mortality risk at the level of the individual (Heidinger et al., 2012). Obviously, the fact that telomere length and the rate of telomere shortening varies with other factors but age decreases its reliability for age determination.

For the measurement of telomere length three different methods have been employed in the literature, potentially leading to biases in the reported values. However, as 39 of the total of 40 studies used telomere restriction fragment length analysis (TRF) analysis, or validated their methods using TRF, which has been described as the original and standard method (Montpetit et al., 2014, Nussey et al., 2014) and only one study used qPCR without TRF validation, we did not distinguish for these different methodologies when assessing the performance of telomere length.

### DNA methylation

DNA methylation is an epigenetic modification, adding a methyl group to the cytosine or adenine residual. DNA methylation often occurs at the C5 position of cytosine residuals that are adjacent to guanidine residuals (CpG site) (Jones, 2012). DNA methylation increases or decreases with age, depending on the position of a specific CpG site (Hannum et al., 2013). Besides age, DNA methylation levels are affected by environmental factors, e.g. sun exposure (Gronniger et al., 2010), life style, e.g. consumption of alcohol and tobacco (Weidner et al., 2014) and diseases, e.g. diabetes (Gu et al., 2013). To improve the accuracy in age determination, DNA methylation levels are often examined in more than one CpG site within one or more genes. Its application to determine age has so far been tried in humans and other mammals (e.g. Polanowski et al., 2014, Zbiec-Piekarska et al., 2015). A more detailed review on this technique and its application can be found in Jarman et al. (2015).

### sjTREC

The thymus rearranges T cell receptors to generate diverse T cells. During this rearrangement, small circles of DNA, signal-joint T cell receptor excision circles (sjTRECs), are being produced (Kong et al., 1998). Carrying sjTRECs, these T cells enter peripheral blood as circulating T cells. Circulating T cells replicate in peripheral blood, but sjTREC does not. sjTREC is consequently diluted with each cell division in peripheral blood (Takeshita et al., 1989). In addition, the ability of the thymus to generate T cells declines with age (Douek et al., 1998), meaning fewer sjTRECs are produced. As a result of the dilution effect in combination with a reduced input from the thymus, sjTREC levels in peripheral blood decline with age. Besides age, sjTREC levels can be reduced by diseases, such as HIV infection (Douek et al., 1998) or the decline with age can be slowed down by diseases such as acute myocardial infarction (Lakhonina et al., 2012). This technique has so far been used to determine age in humans (Cho et al., 2014) and dogs (Ito et al., 2015). More information on this technique can be found in a review by Jarman et al. (2015).

### Racemization

In metabolically inactive tissues, such as teeth and eye lens nuclei, the L-enantiomer (L) of aspartic acid converts to the D-enantiomer (D) over time, a process called racemization (Garde et al., 2010). This racemization occurs at a relatively constant rate throughout the life of an individual (Garde et al., 2010), although the process can be accelerated by high body temperatures (Bada and Brown, 1980). It is possible to estimate the age of an animal when its D/L ratio at birth and the racemization rate are known (Wehmille and Hare, 1971). Racemization has been widely applied to determine age in humans using teeth (Ohtani and Yamamoto, 2005) and has also occasionally been employed in marine mammals using eye lenses (Garde et al., 2007, Garde et al., 2010) and birds using tendons (Hunter et al., 2003). Although the application of racemization has thus mainly been in humans and much less frequently in other taxa, we include this aging technique into our review to evaluate its potential use to a wider range of taxa in the future.

### Otolith ring count

Otoliths are hard structures embedded in the inner ears of bony fishes and are primarily comprised of calcium carbonate (Degens et al., 1969). Growth rings in otoliths are suggested to be formed annually in a large number of fish species (Gooley, 1992, Santana et al., 2009). Otolith ring count has generally been perceived as a highly accurate aging method. However, growth rings may not be deposited every year (Fowler, 1995) and false (non-annual) rings can appear, e.g. due to critical moments in development, such as sex maturity (Khan and Khan, 2009). Nevertheless, otolith ring count has developed into a traditional aging technique in fishes, ages inferred from this technique frequently being assumed to represent "true" age, and subsequently used to validate other aging techniques, such as the below discussed otolithometry and age-length keys.

### Otolithometry

Otoliths grow throughout life without reabsorption (Campana, 1999). The strong linear correlations between both otolith size (i.e. length, breadth and height) and weight with age (Steward et al., 2009) were confirmed in a number of species. Otolithometry therefore was proposed as another potential approach to age fish (Fossen et al., 2003).

### Age-length keys

In addition to otolith ring count and otolithometry, age-length key, which is the correlation between body length and age (class), has also been widely applied in studies in fish. Von Bertalanffy’s growth model is among the most popular models used to describe age-length keys: $L_{t}=L_{\infty}(1-e^{-k(t-t\text{0}\text{)}})$, where *L_t_* is the observed mean length at age *t*, *L_∞_* is the maximum length fish can theoretically achieve, *k* is the growth constant and *t_0_* the theoretical age at zero length (Templeman and Squires, 1956). To ease fitting of growth data to this exponential age-length equation, it is commonly converted into a linear model:

$\ln\left( 1-{L_{t}}/{L_{\infty}} \right)=kt_{0}-kt$.

### Skeletochronology

Similar to growth rings in otoliths, growth rings in bones are formed by seasonal growth and are suggested to increment annually (de Buffrénil and Castanet, 2000, Snover et al., 2011, Friedl and Klump, 1997). The use of growth rings to reconstruct growth history and estimate age of an individual is termed skeletochronology. This technique has mainly been used in reptiles and amphibians, but also in mammals and birds (Sinsch, 2015). As for otolith ring counts, skeletochronology has been perceived as a highly accurate aging method and frequently assumed to represent true age (e.g. Cogălniceanu et al., 2017, Otero et al., 2017, Comas et al., 2016). However, age can be overestimated by noncyclic growth rings introduced by irregular environmental conditions, interrupting the seasonal growth pattern (e.g. elevated ambient temperature that interrupts hibernation (Sinsch et al., 2007)). Age could be underestimated if growth rings are damaged by factors such as bone reabsorption (Bjorndal et al., 1998) and rapprochement of (mostly peripheral) growth rings (Eden et al., 2007). More information on this technique can be found in a review by Sinsch (2015).

**Reference**

BADA, J. L. & BROWN, S. E. 1980. Amino acid recemization in living mammals: biochronological applications. *Trends in Biochemical Sciences,* 5**,** III-V.

BJORNDAL, K. A., BOLTEN, A. B., BENNETT, R. A., JACOBSON, E. R., WRONSKI, T. J., VALESKI, J. J. & ELIAZAR, P. J. 1998. Age and growth in sea turtles: limitations of skeletochronology for demographic studies. *Copeia***,** 23-30.

CAMPANA, S. E. 1999. Chemistry and composition of fish otoliths: Pathways, mechanisms and applications. *Marine Ecology Progress Series,* 188**,** 263-297.

CHO, S., GE, J., SEO, S. B., KIM, K., LEE, H. Y. & LEE, S. D. 2014. Age estimation via quantification of signal-joint T cell receptor excision circles in Koreans. *Legal Medicine,* 16**,** 135-138.

COGĂLNICEANU, D., BĂNCILĂ, R. I., PLĂIAŞU, R., ROŞIORU, D. & MERILÄ, J. 2017. Small-scale spatial and temporal variation of life-history traits of common frogs (Rana temporaria) in sub-Arctic Finland. *Polar Biology,* 40**,** 1581-1592.

COMAS, M., REGUERA, S., ZAMORA–CAMACHO, F., SALVADÓ, H. & MORENO–RUEDA, G. 2016. Comparison of the effectiveness of phalanges vs. humeri and femurs to estimate lizard age with skeletochronology. *Animal Biodiversity and Conservation,* 39**,** 237-240.

DE BUFFRÉNIL, V. & CASTANET, J. 2000. Age estimation by skeletochronology in the Nile monitor (Varanus niloticus), a highly exploited species. *Journal of Herpetology***,** 414-424.

DEGENS, E. T., DEUSER, W. G. & HAEDRICH, R. L. 1969. Molecular structure and composition of fish otoliths. *Marine Biology,* 2**,** 105-113.

DOUEK, D. C., MCFARLAND, R. D., KEISER, P. H., GAGE, E. A., MASSEY, J. M., HAYNES, B. F., POLIS, M. A., HAASE, A. T., FEINBERG, M. B., SULLIVAN, J. L., JAMIESON, B. D., ZACK, J. A., PICKER, L. J. & KOUP, R. A. 1998. Changes in thymic function with age and during the treatment of HIV infection. *Nature,* 396**,** 690-695.

DUNSHEA, G., DUFFIELD, D., GALES, N., HINDELL, M., WELLS, R. S. & JARMAN, S. N. 2011. Telomeres as age markers in vertebrate molecular ecology. *Molecular Ecology Resources,* 11**,** 225-235.

EDEN, C. J., WHITEMAN, H. H., DUOBINIS-GRAY, L. & WISSINGER, S. A. 2007. Accuracy assessment of skeletochronology in the Arizona tiger salamander (Ambystoma tigrinum nebulosum). *Copeia,* 2007**,** 471-477.

FOSSEN, I., ALBERT, O. T. & NILSSEN, E. M. 2003. Improving the precision of ageing assessments for long rough dab by using digitised pictures and otolith measurements. *Fisheries Research,* 60**,** 53-64.

FOWLER, A. 1995. Annulus formation in otoliths of coral reef fish-a review. *In:* SECOR, D., DEAN, J. & CAMPANA, S. (eds.) *Recent developments in fish otolith research.* Columbia: University of South Carolina Press.

FRIEDL, T. W. & KLUMP, G. M. 1997. Some aspects of population biology in the European treefrog, Hyla arborea. *Herpetologica***,** 321-330.

GARDE, E., FRIE, A. K., DUNSHEA, G., HANSEN, S. H., KOVACS, K. M. & LYDERSEN, C. 2010. Harp seal ageing techniques-teeth, aspartic acid racemization, and telomere sequence analysis. *Journal of Mammalogy,* 91**,** 1365-1374.

GARDE, E., HEIDE-JORGENSEN, M. P., HANSEN, S. H., NACHMAN, G. & FORCHHAMMER, M. C. 2007. Age-specific growth and remarkable longevity in narwhals (*Monodon monoceros*) from West Greenland as estimated by aspartic acid racemization. *Journal of Mammalogy,* 88**,** 49-58.

GOOLEY, G. J. 1992. Validation of the use of otoliths to determine the age and growth of Murray cod, *Maccullochella peelii* (Mitchell) (Percichthyidae), in Lake Charlegrark, western Victoria. *Australian Journal of Marine & Freshwater Research,* 43**,** 1091-1102.

GRONNIGER, E., WEBER, B., HEIL, O., PETERS, N., STAB, F., WENCK, H., KORN, B., WINNEFELD, M. & LYKO, F. 2010. Aging and chronic sun exposure cause distinct epigenetic changes in human skin. *PLoS Genet,* 6**,** e1000971.

GU, T., GU, H. F., HILDING, A., SJÖHOLM, L. K., OSTENSON, C.-G., EKSTRÖM, T. J. & BRISMAR, K. 2013. Increased DNA methylation levels of the insulin-like growth factor binding protein 1 gene are associated with type 2 diabetes in Swedish men. *Clin Epigenetics,* 5**,** 21.

HANNUM, G., GUINNEY, J., ZHAO, L., ZHANG, L., HUGHES, G., SADDA, S., KLOTZLE, B., BIBIKOVA, M., FAN, J.-B., GAO, Y., DECONDE, R., CHEN, M., RAJAPAKSE, I., FRIEND, S., IDEKER, T. & ZHANG, K. 2013. Genome-wide Methylation Profiles Reveal Quantitative Views of Human Aging Rates. *Molecular Cell,* 49**,** 359-367.

HAUSSMANN, M. F., WINKLER, D. W. & VLECK, C. M. 2005. Longer telomeres associated with higher survival in birds. *Biology letters,* 1**,** 212-214.

HEIDINGER, B. J., BLOUNT, J. D., BONER, W., GRIFFITHS, K., METCALFE, N. B. & MONAGHAN, P. 2012. Telomere length in early life predicts lifespan. *Proceedings of the National Academy of Sciences,* 109**,** 1743-1748.

HUNTER, E., METCALFE, J. D. & REYNOLDS, J. D. 2003. Migration route and spawning area fidelity by North Sea plaice. *Proceedings of the Royal Society of London B: Biological Sciences,* 270**,** 2097-2103.

ITO, G., YOSHIMURA, K. & MOMOI, Y. 2015. Gene analysis of signal-joint T cell receptor excision circles and their relationship to age in dogs. *Veterinary Immunology and Immunopathology,* 166**,** 1-7.

JARMAN, S. N., POLANOWSKI, A. M., FAUX, C. E., ROBBINS, J., DE PAOLI-ISEPPI, R., BRAVINGTON, M. & DEAGLE, B. E. 2015. Molecular biomarkers for chronological age in animal ecology. *Molecular Ecology,* 24**,** 4826-4847.

JONES, P. A. 2012. Functions of DNA methylation: islands, start sites, gene bodies and beyond. *Nature Reviews Genetics,* 13**,** 484-492.

KHAN, M. A. & KHAN, S. 2009. Comparison of age estimates from scale, opercular bone, otolith, vertebrae and dorsal fin ray in Labeo rohita (Hamilton), Catla catla (Hamilton) and Channa marulius (Hamilton). *Fisheries Research,* 100**,** 255-259.

KONG, F., CHEN-LO, H. C. & COOPER, M. D. 1998. Thymic function can be accurately monitored by the level of recent T cell emigrants in the circulation. *Immunity,* 8**,** 97-104.

LAKHONINA, N., GOLOVIZNIN, M., DONETSKOVA, A., NIKONOVA, M., YARILIN, A., BULDAKOVA, Y. & TEKTOVA, A. 2012. T cell receptor rearrangement excision circles (TREC) study as an approach to "in vivo" thymus gland function investigation. *Arthritis Research & Therapy,* 14**,** P37-P37.

MONTPETIT, A. J., ALHAREERI, A. A., MONTPETIT, M., STARKWEATHER, A. R., ELMORE, L. W., FILLER, K., MOHANRAJ, L., BURTON, C. W., MENZIES, V. S. & LYON, D. E. 2014. Telomere length: a review of methods for measurement. *Nursing research,* 63**,** 289.

NUSSEY, D. H., BAIRD, D., BARRETT, E., BONER, W., FAIRLIE, J., GEMMELL, N., HARTMANN, N., HORN, T., HAUSSMANN, M. & OLSSON, M. 2014. Measuring telomere length and telomere dynamics in evolutionary biology and ecology. *Methods in Ecology and Evolution,* 5**,** 299-310.

OHTANI, S. & YAMAMOTO, T. 2005. Strategy for the estimation of chronological age using the aspartic acid racemization method with special reference to coefficient of correlation between D/L ratios and ages. *Journal of Forensic Sciences,* 50**,** 1020-1027.

OTERO, M., BARAQUET, M., POLLO, F., GRENAT, P., SALAS, N. & MARTINO, A. 2017. Sexual Size Dimorphism in Relation to Age and Growth in Hypsiboas cordobae (Anura: Hylidae) from Córdoba, Argentina. *Herpetological Conservation and Biology,* 12**,** 141-148.

POLANOWSKI, A. M., ROBBINS, J., CHANDLER, D. & JARMAN, S. N. 2014. Epigenetic estimation of age in humpback whales. *Molecular Ecology Resources,* 14**,** 976-987.

SANTANA, F. M., MORIZE, E., CLAVIER, J. & LESSA, R. 2009. Otolith micro- and macrostructure analysis to improve accuracy of growth parameter estimation for white mullet Mugil curema. *Aquatic Biology,* 7**,** 199-206.

SINSCH, U. 2015. Skeletochronological assessment of demographic life-history traits in amphibians. *The Herpetological Journal,* 25**,** 5-13.

SINSCH, U., OROMI, N. & SANUY, D. 2007. Growth marks in natterjack toad (Bufo calamita) bones: histological correlates of hibernation and aestivation periods. *The Herpetological Journal,* 17**,** 129-137.

SNOVER, M. L., HOHN, A. A., GOSHE, L. R. & BALAZS, G. H. 2011. Validation of annual skeletal marks in green sea turtles Chelonia mydas using tetracycline labeling. *Aquatic Biology,* 12**,** 197-204.

STEWARD, C. A., DEMARIA, K. D. & SHENKER, J. M. 2009. Using otolith morphometrics to quickly and inexpensively predict age in the gray angelfish (Pomacanthus arcuatus). *Fisheries Research,* 99**,** 123-129.

TAKESHITA, S., TODA, M. & YAMAGISHI, H. 1989. Excision products of the T cell receptor gene support a progressive rearrangement model of the alpha/delta locus. *The EMBO journal,* 8**,** 3261.

TEMPLEMAN, W. & SQUIRES, H. J. 1956. Relationship of otolith lengths and weights in the haddock Melanogrammus aeglefinus (L.) to the rate of growth of the fish. *Canadian Journal of Fisheries and Aquatic Sciences,* 13**,** 467-487.

WEHMILLE, J. & HARE, P. E. 1971. Racemization of amino acids in marine sediments. *Science,* 173**,** 907-911.

WEIDNER, C. I., LIN, Q., KOCH, C. M., EISELE, L., BEIER, F., ZIEGLER, P., BAUERSCHLAG, D. O., JÖCKEL, K.-H., ERBEL, R. & MÜHLEISEN, T. W. 2014. Aging of blood can be tracked by DNA methylation changes at just three CpG sites. *Genome biology,* 15**,** R24.

ZBIEC-PIEKARSKA, R., SPOLNICKA, M., KUPIEC, T., MAKOWSKA, Z., SPAS, A., PARYS-PROSZEK, A., KUCHARCZYK, K., PLOSKI, R. & BRANICKI, W. 2015. Examination of DNA methylation status of the ELOVL2 marker may be useful for human age prediction in forensic science. *Forensic Science International-Genetics,* 14**,** 161-167.
