# Supplementary material for: The adequacy of aging techniques in vertebrates for rapid estimation of population mortality rates from age distributions
Source: Ecol Evol. 2018 Dec 27;9(3):1394–402. doi: 10.1002/ece3.4854 (PMC6374686; doi:10.1002/ece3.4854)
Supplement: Supplementary file 4 [file ECE3-9-1394-s004.docx]

**Appendix S4.** $\boldsymbol{R}^{\mathbf{2}}$ **and** $\mathbf{|}\boldsymbol{\beta/\sigma}\mathbf{|}$ **for case studies employing eight different aging techniques (telomere length, racemization, DNA methylation, signal-joint T-cell Recombination Excision Circle (sjTREC), otolith ring count, otolithometry, age-length keys and skeletochronology) in vertebrates.**

| **Indicator** | **Taxa** | **Species** | **Latin name** | $\boldsymbol{R}^{\mathbf{2}}$ | $\mathbf{\vert}\boldsymbol{\beta/\sigma}\mathbf{\vert}$ | **Reference** |
| --- | --- | --- | --- | --- | --- | --- |
| Telomere length | Human | Human | Homo sapiens | 0.56 | 0.08 | ([Takasaki et al., 2003](#_ENREF_108)) |
|  |  | Human | Homo sapiens | 0.69 | 0.07 | ([Tsuji et al., 2002](#_ENREF_110)) |
|  |  | Human | Homo sapiens | 0.09 | 0.04 | ([Unryn et al., 2005](#_ENREF_113)) |
|  |  | Human | Homo sapiens | 0.26 | 0.03 | ([Lahnert, 2005](#_ENREF_52)) |
|  |  | Human | Homo sapiens | 0.31 | 0.02 | ([Unryn et al., 2005](#_ENREF_113)) |
|  |  | Human | Homo sapiens | 0.22 | 0.02 | ([Lahnert, 2005](#_ENREF_52)) |
|  |  | Human | Homo sapiens | 0.18 | 0.01 | ([Allsopp et al., 1992](#_ENREF_1)) |
|  |  | Human | Homo sapiens | 0.30 | 0.02 | ([Lindsey et al., 1991](#_ENREF_55)) |
|  |  | Human | Homo sapiens | 0.29 | 0.02 | ([Melk et al., 2000](#_ENREF_61)) |
|  |  | Human | Homo sapiens | 0.25 | 0.03 | ([Hastie et al., 1990](#_ENREF_31)) |
|  |  | Human | Homo sapiens | 0.40 | 0.03 | ([Furugori et al., 2000](#_ENREF_24)) |
|  |  | Human | Homo sapiens | 0.36 | 0.04 | ([Kang et al., 2002](#_ENREF_48)) |
|  |  | Human | Homo sapiens | 0.60 | 0.04 | ([Yang et al., 2001](#_ENREF_119)) |
|  |  | Human | Homo sapiens | 0.35 | 0.05 | ([Wiemann et al., 2002](#_ENREF_118)) |
|  | Mammal | Dog | Canis familiaris | 0.02 | 0.04 | ([Nasir et al., 2001](#_ENREF_65)) |
|  |  | Dog | Canis familiaris | 0.02 | 0.03 | ([Nasir et al., 2001](#_ENREF_65)) |
|  |  | Donkey | Equus asinus | 0.17 | 0.05 | ([Argyle et al., 2003](#_ENREF_3)) |
|  |  | Cynomolgus monkey | Macaca fascicularis | 0.27 | 0.06 | ([Lee et al., 2002](#_ENREF_54)) |
|  |  | Dog | Canis familiaris | 0.47 | 0.42 | ([Yazawa et al., 2001](#_ENREF_120)) |
|  |  | Horse | Equus caballus | 0.64 | 0.15 | ([Katepalli et al., 2008](#_ENREF_49)) |
|  |  | Japanese Black cattle | Bos taurus | 0.62 | 0.24 | ([Miyashita et al., 2002](#_ENREF_62)) |
|  |  | Sheep | Ovis aries | 0.39 | 0.42 | ([Shiels et al., 1999](#_ENREF_104)) |
|  | Bird | Zebra finch | Taeniopygia guttata | 0.54 | 1.60 | ([Haussmann and Vleck, 2002](#_ENREF_35)) |
|  |  | Zebra finch | Taeniopygia guttata | 0.82 | 1.52 | ([Haussmann and Mauck, 2008](#_ENREF_34)) |
|  |  | Tree swallow | Tachycineta bicolor | 0.34 | 0.36 | ([Haussmann et al., 2003b](#_ENREF_37)) |
|  |  | Adelie penguin | Pygoscelis adeliae | 0.55 | 0.30 | ([Haussmann et al., 2003b](#_ENREF_37)) |
|  |  | European shag | Phalacrocorax aristotelis | 0.00 | 0.01 | ([Hall et al., 2004](#_ENREF_29)) |
|  |  | Common tern | Sterna hirundo | 0.61 | 0.17 | ([Haussmann et al., 2003a](#_ENREF_36)) |
|  |  | Leach’s storm-petrel | Oceanodroma leucorhoa | 0.66 | 0.14 | ([Haussmann et al., 2003b](#_ENREF_37)) |
|  |  | Great frigatebird | Fregata minor | 0.74 | 0.10 | ([Juola et al., 2006](#_ENREF_47)) |
|  |  | Kakapo | Strigops habroptila | 0.02 | 0.01 | ([Horn et al., 2011](#_ENREF_41)) |
|  |  | Kakapo | Strigops habroptila | 0.00 | 0.01 | ([Horn et al., 2011](#_ENREF_41)) |
|  |  | Blue-footed booby | Sula nebouxii | 0.00 | 0.00 | ([Foote, 2008](#_ENREF_19)) |
|  |  | American redstart | Setophaga ruticilla | 0.29 | 0.00 | ([Angelier et al., 2013](#_ENREF_2)) |
|  |  | Thick-billed murre | Uria lomvia | 0.10 | 0.04 | ([Young et al., 2013](#_ENREF_121)) |
|  |  | Alpine swift | Apus melba | 0.03 | 0.04 | ([Bize et al., 2009](#_ENREF_5)) |
|  |  | Wandering albatross | Diomedea exulans | 0.00 | 0.01 | ([Hall et al., 2004](#_ENREF_29)) |
|  |  | Giant petrel | Macronectes giganteus | 0.62 | 0.10 | ([Foote, 2008](#_ENREF_19)) |
|  |  | Giant petrel | Macronectes halli | 0.43 | 0.10 | ([Foote, 2008](#_ENREF_19)) |
|  |  | Thick-billed murre | Uria lomvia | 0.41 | 0.11 | ([Young et al., 2013](#_ENREF_121)) |
|  |  | Dunlin | Calidris alpina | 0.12 | 0.11 | ([Pauliny et al., 2006](#_ENREF_90)) |
|  |  | Sand martin | Riparia riparia | 0.34 | 0.29 | ([Pauliny et al., 2006](#_ENREF_90)) |
|  | Fish | Japanese black porgy | Acanthopagrus schlegeli | 0.33 | 0.66 | ([Tsui, 2005](#_ENREF_109)) |
|  |  | Medaka | Oryzias latipes | 0.33 | 0.64 | ([Hatakeyama et al., 2008](#_ENREF_32)) |
|  |  | Common carp | Cyprinus carpio | 0.80 | 0.54 | ([Izzo, 2010](#_ENREF_46)) |
|  |  | Common carp | Cyprinus carpio | 0.73 | 0.51 | ([Izzo, 2010](#_ENREF_46)) |
|  |  | Japanese black porgy | Acanthopagrus schlegeli | 0.06 | 0.35 | ([Tsui, 2005](#_ENREF_109)) |
|  |  | Common carp | Cyprinus carpio | 0.09 | 0.12 | ([Izzo, 2010](#_ENREF_46)) |
|  |  | Common carp | Cyprinus carpio | 0.00 | 0.02 | ([Izzo, 2010](#_ENREF_46)) |
|  |  | European sea bass | Dicentrarchus labrax | 0.03 | 0.01 | ([Horn et al., 2008](#_ENREF_40)) |
|  |  | Golden perch | Macquaria ambigua | 0.01 | 0.07 | ([Izzo, 2010](#_ENREF_46)) |
|  |  | Mangrove red snapper | Lutjanus argentimaculatus | 0.15 | 0.34 | ([Tsui, 2005](#_ENREF_109)) |
|  |  | Bluefin leatherjacket | Thammaconus degeni | 0.75 | 0.43 | ([Izzo, 2010](#_ENREF_46)) |
|  |  | Mangrove red snapper | Lutjanus argentimaculatus | 0.25 | 0.47 | ([Tsui, 2005](#_ENREF_109)) |
|  |  | Australasian snapper | Chrysophyrys auratus | 0.80 | 0.50 | ([Izzo, 2010](#_ENREF_46)) |
|  |  | Mangrove red snapper | Lutjanus argentimaculatus | 0.38 | 0.51 | ([Tsui, 2005](#_ENREF_109)) |
|  |  | Japanese black porgy | Acanthopagrus schlegeli | 0.21 | 0.56 | ([Tsui, 2005](#_ENREF_109)) |
|  |  | Sand flathead | Platycephalus bassensis | 0.74 | 0.62 | ([Izzo, 2010](#_ENREF_46)) |
|  |  | Japanese black porgy | Acanthopagrus schlegeli | 0.18 | 0.81 | ([Tsui, 2005](#_ENREF_109)) |
|  |  | Bluespotted goatfish | Upeneichthys vlamingii | 0.85 | 1.02 | ([Izzo, 2010](#_ENREF_46)) |
|  | Reptile | Water python | Liasis fuscus | 0.00 | 0.02 | ([Ujvari and Madsen, 2009](#_ENREF_112)) |
|  |  | Water python | Liasis fuscus | 0.01 | 0.02 | ([Ujvari and Madsen, 2009](#_ENREF_112)) |
|  |  | Loggerhead turtle | Caretta caretta | 0.09 | 0.02 | ([Hatase et al., 2008](#_ENREF_33)) |
|  |  | Loggerhead turtle | Caretta caretta | 0.00 | 0.01 | ([Hatase et al., 2008](#_ENREF_33)) |
|  |  | Sand lizard | Lacerta agilis | 0.12 | 0.37 | ([Olsson et al., 2011](#_ENREF_84)) |
|  |  | Garter snake | Thamnophis elegans | 0.73 | 0.46 | ([Bronikowski, 2008](#_ENREF_8)) |
|  | Shark | Port Jackson shark | Heterodontus portusjacksoni | 0.03 | 0.04 | ([Izzo, 2010](#_ENREF_46)) |
|  |  | Port Jackson shark | Heterodontus portusjacksoni | 0.10 | 0.05 | ([Izzo, 2010](#_ENREF_46)) |
| DNA methylation | Human | Human | Homo sapiens | 0.82 | 0.15 | ([Lee et al., 2015](#_ENREF_53)) |
|  |  | Human | Homo sapiens | 0.95 | 0.21 | ([Bekaert et al., 2015](#_ENREF_4)) |
|  |  | Human | Homo sapiens | 0.86 | 0.15 | ([Zbiec-Piekarska et al., 2015](#_ENREF_122)) |
|  |  | Human | Homo sapiens | 0.69 | 0.13 | ([Bocklandt et al., 2011](#_ENREF_7)) |
|  |  | Human | Homo sapiens | 0.93 | 0.26 | ([Hannum et al., 2013](#_ENREF_30)) |
|  |  | Human | Homo sapiens | 0.06 | 0.02 | ([Christensen et al., 2009](#_ENREF_13)) |
|  |  | Human | Homo sapiens | 0.98 | 0.23 | ([Weidner et al., 2014](#_ENREF_116)) |
|  |  | Human | Homo sapiens | 0.65 | 0.08 | ([Koch and Wagner, 2011](#_ENREF_51)) |
|  |  | Human | Homo sapiens | 0.79 | 0.15 | ([Horvath et al., 2014](#_ENREF_43)) |
|  | Mammal | Mouse | Mus musculus | 0.93 | 0.27 | ([Maegawa et al., 2010](#_ENREF_56)) |
|  |  | Bonobo | Pan paniscus | 0.71 | 0.37 | ([Horvath, 2013](#_ENREF_42)) |
|  |  | Gorilla | Gorilla beringei graueri;  Gorilla gorilla gorilla | 0.00 | 0.00 | ([Horvath, 2013](#_ENREF_42)) |
|  |  | Humpback whale | Megaptera novaeangliae | 0.79 | 0.21 | ([Polanowski et al., 2014](#_ENREF_93)) |
| sjTREC | Human | Human | Homo sapiens | 0.67 | 2.38 | ([Ou et al., 2011](#_ENREF_87)) |
|  |  | Human | Homo sapiens | 0.65 | 0.18 | ([Cho et al., 2014](#_ENREF_12)) |
|  |  | Human | Homo sapiens | 0.76 | 0.76 | ([Ou et al., 2012](#_ENREF_86)) |
|  |  | Human | Homo sapiens | 0.77 | 0.76 | ([Qu et al., 2013](#_ENREF_95)) |
|  |  | Human | Homo sapiens | 0.84 | 0.10 | ([Zubakov et al., 2010](#_ENREF_123)) |
|  |  | Human | Homo sapiens | 0.85 | 0.11 | ([Douek et al., 1998](#_ENREF_17)) |
|  | Mammal | Dog | Canis familiaris | 0.00 | 0.00 | ([Ito et al., 2015](#_ENREF_45)) |
| Racemization | Human | Human | Homo sapiens | 0.96 | 2.39 | ([Ohtani, 1994](#_ENREF_73)) |
|  |  | Human | Homo sapiens | 0.88 | 1.58 | ([Ohtani, 1994](#_ENREF_73)) |
|  |  | Human | Homo sapiens | 0.98 | 1.42 | ([Ohtani and Yamamoto, 1991](#_ENREF_80)) |
|  |  | Human | Homo sapiens | 0.99 | 0.91 | ([Ohtani and Yamamoto, 2010](#_ENREF_82)) |
|  |  | Human | Homo sapiens | 0.99 | 0.82 | ([Ohtani et al., 1995](#_ENREF_77)) |
|  |  | Human | Homo sapiens | 0.99 | 0.81 | ([Ohtani et al., 1995](#_ENREF_77)) |
|  |  | Human | Homo sapiens | 0.86 | 0.80 | ([Ohtani, 1994](#_ENREF_73)) |
|  |  | Human | Homo sapiens | 0.99 | 0.44 | ([Ohtani et al., 1995](#_ENREF_77)) |
|  |  | Human | Homo sapiens | 0.99 | 0.53 | ([Ohtani et al., 1995](#_ENREF_77)) |
|  |  | Human | Homo sapiens | 0.87 | 0.80 | ([Ohtani, 1994](#_ENREF_73)) |
|  |  | Human | Homo sapiens | 0.99 | 0.78 | ([Ohtani and Yamamoto, 2010](#_ENREF_82)) |
|  |  | Human | Homo sapiens | 0.98 | 0.61 | ([Ohtani et al., 1995](#_ENREF_77)) |
|  |  | Human | Homo sapiens | 0.99 | 0.70 | ([Ohtani et al., 1995](#_ENREF_77)) |
|  |  | Human | Homo sapiens | 0.97 | 0.69 | ([Ohtani and Yamamoto, 2010](#_ENREF_82)) |
|  |  | Human | Homo sapiens | 0.99 | 0.65 | ([Ohtani and Yamamoto, 1987](#_ENREF_79)) |
|  |  | Human | Homo sapiens | 0.99 | 0.64 | ([Ohtani and Yamamoto, 1992](#_ENREF_81)) |
|  |  | Human | Homo sapiens | 0.98 | 0.63 | ([Ohtani and Yamamoto, 2010](#_ENREF_82)) |
|  |  | Human | Homo sapiens | 0.98 | 0.56 | ([Ohtani, 1995](#_ENREF_74)) |
|  |  | Human | Homo sapiens | 0.98 | 0.43 | ([Ohtani et al., 1995](#_ENREF_77)) |
|  |  | Human | Homo sapiens | 0.68 | 0.52 | ([Ohtani, 1994](#_ENREF_73)) |
|  |  | Human | Homo sapiens | 0.98 | 0.50 | ([Ohtani and Yamamoto, 2011](#_ENREF_83)) |
|  |  | Human | Homo sapiens | 0.97 | 0.29 | ([Ohtani et al., 1995](#_ENREF_77)) |
|  |  | Human | Homo sapiens | 0.98 | 0.44 | ([Fu et al., 1995](#_ENREF_22)) |
|  |  | Human | Homo sapiens | 0.98 | 0.43 | ([Ohtani, 1995](#_ENREF_74)) |
|  |  | Human | Homo sapiens | 0.98 | 0.42 | ([Ritz et al., 1993](#_ENREF_98)) |
|  |  | Human | Homo sapiens | 0.98 | 0.42 | ([Ogino et al., 1985](#_ENREF_72)) |
|  |  | Human | Homo sapiens | 0.98 | 0.41 | ([Ohtani et al., 2004](#_ENREF_78)) |
|  |  | Human | Homo sapiens | 0.95 | 0.39 | ([Ohtani and Yamamoto, 2011](#_ENREF_83)) |
|  |  | Human | Homo sapiens | 0.98 | 0.36 | ([Ritz et al., 1996](#_ENREF_101)) |
|  |  | Human | Homo sapiens | 0.99 | 0.35 | ([Ohtani and Yamamoto, 2010](#_ENREF_82)) |
|  |  | Human | Homo sapiens | 0.97 | 0.33 | ([Ohtani et al., 2002](#_ENREF_76)) |
|  |  | Human | Homo sapiens | 0.98 | 0.32 | ([Ritz-Timme et al., 2003](#_ENREF_97)) |
|  |  | Human | Homo sapiens | 0.97 | 0.29 | ([Ohtani, 1995](#_ENREF_74)) |
|  |  | Human | Homo sapiens | 0.96 | 0.27 | ([van den Oetelaar and Hoenders, 1989](#_ENREF_114)) |
|  |  | Human | Homo sapiens | 0.98 | 0.25 | ([Ritz et al., 1994](#_ENREF_100)) |
|  |  | Human | Homo sapiens | 0.95 | 0.25 | ([Ohtani et al., 2002](#_ENREF_76)) |
|  |  | Human | Homo sapiens | 0.95 | 0.25 | ([Rajkumari et al., 2013](#_ENREF_96)) |
|  |  | Human | Homo sapiens | 0.97 | 0.24 | ([Ohtani and Yamamoto, 2011](#_ENREF_83)) |
|  |  | Human | Homo sapiens | 0.95 | 0.24 | ([Ohtani et al., 2002](#_ENREF_76)) |
|  |  | Human | Homo sapiens | 0.96 | 0.24 | ([Helfman and Bada, 1976](#_ENREF_38)) |
|  |  | Human | Homo sapiens | 0.61 | 0.22 | ([Shapiro et al., 1991](#_ENREF_103)) |
|  |  | Human | Homo sapiens | 0.92 | 0.22 | ([Ritz et al., 1994](#_ENREF_100)) |
|  |  | Human | Homo sapiens | 1.00 | 0.18 | ([Ohtani and Yamamoto, 2010](#_ENREF_82)) |
|  |  | Human | Homo sapiens | 0.92 | 0.17 | ([Ritz et al., 1990](#_ENREF_99)) |
|  |  | Human | Homo sapiens | 0.87 | 0.14 | ([Ohtani et al., 2002](#_ENREF_76)) |
|  |  | Human | Homo sapiens | 0.89 | 0.14 | ([Man et al., 1983](#_ENREF_57)) |
|  |  | Human | Homo sapiens | 0.90 | 0.14 | ([Verzijl et al., 2000](#_ENREF_115)) |
|  |  | Human | Homo sapiens | 0.94 | 0.14 | ([Pfeiffer et al., 1995a](#_ENREF_91)) |
|  |  | Human | Homo sapiens | 0.73 | 0.12 | ([Ohtani et al., 1998](#_ENREF_75)) |
|  |  | Human | Homo sapiens | 0.78 | 0.10 | ([Ohtani et al., 2002](#_ENREF_76)) |
|  |  | Human | Homo sapiens | 0.83 | 0.09 | ([Masters et al., 1977](#_ENREF_60)) |
|  |  | Human | Homo sapiens | 0.81 | 0.09 | ([Maroudas et al., 1998](#_ENREF_59)) |
|  |  | Human | Homo sapiens | 0.77 | 0.08 | ([Shimoyama and Harada, 1984](#_ENREF_105)) |
|  |  | Human | Homo sapiens | 0.58 | 0.07 | ([Ohtani et al., 2002](#_ENREF_76)) |
|  |  | Human | Homo sapiens | 0.61 | 0.06 | ([Verzijl et al., 2000](#_ENREF_115)) |
|  |  | Human | Homo sapiens | 0.55 | 0.06 | ([Ohtani et al., 2002](#_ENREF_76)) |
|  |  | Human | Homo sapiens | 0.76 | 0.06 | ([Fujii et al., 1999](#_ENREF_23)) |
|  |  | Human | Homo sapiens | 0.71 | 0.06 | ([Pfeiffer et al., 1995b](#_ENREF_92)) |
|  | Mammal | Fin whale and Narwhals | Balaenoptera physalus and Monodon monoceros | 0.90 | 0.39 | ([Garde et al., 2007](#_ENREF_26)) |
|  |  | Harp seal | Pagophilus groenlandicus | 0.93 | 0.36 | ([Garde et al., 2010](#_ENREF_25)) |
|  | Bird | Eastern bluebird | Sialia sialis | 0.45 | 0.98 | ([Hunter, 1989](#_ENREF_44)) |
|  |  | Brown pelican | Pelecanus occidentalis | 0.53 | 0.17 | ([Hunter, 1989](#_ENREF_44)) |
|  |  | Western gull | Larus occidentalis | 0.02 | 0.04 | ([Hunter, 1989](#_ENREF_44)) |
| Otolithometry, OW | Fish | Five-lined snapper | Lutjanus quinquelineatus | 0.91 | 75.20 | ([Newman et al., 1996](#_ENREF_71)) |
|  |  | Brown-striped red snapper | Lutjanus vitta | 0.80 | 62.09 | ([Newman et al., 2000a](#_ENREF_68)) |
|  |  | Brown-striped red snapper | Lutjanus vitta | 0.84 | 53.15 | ([Newman et al., 2000a](#_ENREF_68)) |
|  |  | Yellow-banded snapper | Lutjanus adetii | 0.81 | 50.38 | ([Newman et al., 1996](#_ENREF_71)) |
|  |  | Spanish flag snapper | Lutjanus carponotatus | 0.68 | 31.61 | ([Newman et al., 2000a](#_ENREF_68)) |
|  |  | Spanish flag snapper | Lutjanus carponotatus | 0.68 | 26.67 | ([Newman et al., 2000a](#_ENREF_68)) |
|  |  | Deepsea jewfish | Glaucosoma buergeri | 0.91 | 8.01 | ([Newman, 2002a](#_ENREF_66)) |
|  |  | Emperor red snapper | Lutjanus sebae | 0.88 | 6.40 | ([Newman and Dunk, 2002](#_ENREF_70)) |
|  |  | Emperor red snapper | Lutjanus sebae | 0.85 | 6.29 | ([Newman and Dunk, 2002](#_ENREF_70)) |
|  |  | Malabar blood snapper | Lutjanus malabaricus | 0.92 | 6.24 | ([Newman, 2002b](#_ENREF_67)) |
|  |  | Emperor red snapper | Lutjanus sebae | 0.72 | 0.47 | ([Newman et al., 2000b](#_ENREF_69)) |
|  |  | Crimson snapper | Lutjanus erythropterus | 0.49 | 0.41 | ([Newman et al., 2000b](#_ENREF_69)) |
|  |  | Malabar blood snapper | Lutjanus malabaricus | 0.73 | 0.29 | ([Newman et al., 2000b](#_ENREF_69)) |
| Otolithometry, OL |  | Crimson snapper | Lutjanus erythropterus | 0.86 | 7.85 | ([Newman et al., 2000b](#_ENREF_69)) |
|  |  | Malabar blood snapper | Lutjanus malabaricus | 0.90 | 4.32 | ([Newman et al., 2000b](#_ENREF_69)) |
|  |  | Emperor red snapper | Lutjanus sebae | 0.89 | 4.02 | ([Newman et al., 2000b](#_ENREF_69)) |
|  |  | Five-lined snapper | Lutjanus quinquelineatus | 0.53 | 1.20 | ([Newman et al., 1996](#_ENREF_71)) |
|  |  | Yellow-banded snapper | Lutjanus adetii | 0.35 | 0.95 | ([Newman et al., 1996](#_ENREF_71)) |
|  |  | Brown-striped red snapper | Lutjanus vitta | 0.45 | 0.89 | ([Newman et al., 2000a](#_ENREF_68)) |
|  |  | Spanish flag snapper | Lutjanus carponotatus | 0.28 | 0.52 | ([Newman et al., 2000a](#_ENREF_68)) |
|  |  | Deepsea jewfish | Glaucosoma buergeri | 0.84 | 0.45 | ([Newman, 2002a](#_ENREF_66)) |
|  |  | Malabar blood snapper | Lutjanus malabaricus | 0.71 | 0.35 | ([Newman, 2002b](#_ENREF_67)) |
|  |  | Emperor red snapper | Lutjanus sebae | 0.34 | 0.34 | ([Newman and Dunk, 2002](#_ENREF_70)) |
| Otolithometry, OH |  | Emperor red snapper | Lutjanus sebae | 0.85 | 3.94 | ([Newman and Dunk, 2002](#_ENREF_70)) |
|  |  | Emperor red snapper | Lutjanus sebae | 0.87 | 3.81 | ([Newman and Dunk, 2002](#_ENREF_70)) |
|  |  | Deepsea jewfish | Glaucosoma buergeri | 0.91 | 3.56 | ([Newman, 2002a](#_ENREF_66)) |
|  |  | Malabar blood snapper | Lutjanus malabaricus | 0.85 | 2.96 | ([Newman, 2002b](#_ENREF_67)) |
| Otolithometry, OB |  | Yellow-banded snapper | Lutjanus adetii | 0.46 | 2.00 | ([Newman et al., 1996](#_ENREF_71)) |
|  |  | Brown-striped red snapper | Lutjanus vitta | 0.54 | 1.81 | ([Newman et al., 2000a](#_ENREF_68)) |
|  |  | Five-lined snapper | Lutjanus quinquelineatus | 0.56 | 1.62 | ([Newman et al., 1996](#_ENREF_71)) |
|  |  | Spanish flag snapper | Lutjanus carponotatus | 0.32 | 1.06 | ([Newman et al., 2000a](#_ENREF_68)) |
|  |  | Malabar blood snapper | Lutjanus malabaricus | 0.73 | 0.69 | ([Newman, 2002b](#_ENREF_67)) |
|  |  | Deepsea jewfish | Glaucosoma buergeri | 0.77 | 0.66 | ([Newman, 2002a](#_ENREF_66)) |
|  |  | Emperor red snapper | Lutjanus sebae | 0.32 | 0.51 | ([Newman and Dunk, 2002](#_ENREF_70)) |
|  |  | Emperor red snapper | Lutjanus sebae | 0.77 | 0.32 | ([Newman et al., 2000b](#_ENREF_69)) |
|  |  | Crimson snapper | Lutjanus erythropterus | 0.52 | 0.23 | ([Newman et al., 2000b](#_ENREF_69)) |
|  |  | Malabar blood snapper | Lutjanus malabaricus | 0.75 | 0.19 | ([Newman et al., 2000b](#_ENREF_69)) |
| Otolith ring count | Fish | Murray cod | Maccullochella peelii | 1.00 | Inf | ([Gooley, 1992](#_ENREF_28)) |
|  |  | Crimson snapper | Lutjanus. erythropterus | 0.95 | 3.84 | ([Cappo et al., 2000](#_ENREF_10)) |
|  |  | Carpenter seabream | Argyrozona argyrozona | 1.00 | 3.34 | ([Brouwer and Griffiths, 2004](#_ENREF_9)) |
|  |  | Australasian snapper | Pagrus auratus | 0.99 | 2.46 | ([Francis et al., 1992](#_ENREF_20)) |
|  |  | John's snapper | Lutjanus. johnii | 0.89 | 2.38 | ([Cappo et al., 2000](#_ENREF_10)) |
|  |  | Japanese black porgy | Acanthopagrus schlegeli | 0.73 | 2.27 | ([Tsui, 2005](#_ENREF_109)) |
|  |  | Mangrove red snapper | Lutjanus argentimaculatus | 0.86 | 1.96 | ([Tsui, 2005](#_ENREF_109)) |
|  |  | Emperor red snapper | Lutjanus. sebae | 0.60 | 1.27 | ([Cappo et al., 2000](#_ENREF_10)) |
| Age-length key | Fish | Shortnose greeneye | Chlorophthalmus agassizii Bonaparte | 0.92 | 12.58 | ([D'Onghia et al., 2006](#_ENREF_14)) |
|  |  | Corvina reina | Cynoscion albus | 0.92 | 8.62 | ([Mug-Villanueva et al., 1994](#_ENREF_64)) |
|  |  | Myers' icefish | Chionodraco myersi | 0.90 | 7.55 | ([Morales-Nin et al., 2000](#_ENREF_63)) |
|  |  | Greater amberjack | Seriola dumerili | 0.79 | 5.48 | ([Manooch Iii and Potts, 1997](#_ENREF_58)) |
|  |  | Vermilion snapper | Rhomboplites aurorubens | 0.73 | 5.07 | ([Potts et al., 1998](#_ENREF_94)) |
|  |  | Red snapper | Lutjanus campechanus | 0.62 | 3.78 | ([White and Palmer, 2004](#_ENREF_117)) |
|  |  | Myers' icefish | Chionodraco myersi | 0.76 | 2.89 | ([Morales-Nin et al., 2000](#_ENREF_63)) |
|  |  | Otolithes ruber | Tigertooth croaker | 0.86 | 2.73 | ([Gh et al., 2012](#_ENREF_27)) |
|  |  | Cadenat's rockfish | Scorpaena loppei | 0.56 | 1.93 | ([Ordines et al., 2012](#_ENREF_85)) |
|  |  | Namibian silver kob | Argyrosomus inodorus | 0.63 | 1.85 | ([Kirchner and Voges, 1999](#_ENREF_50)) |
|  |  | Sharpsnout seabream | Diplodus puntazzo | 0.84 | 1.34 | ([Domínguez-Seoane et al., 2006](#_ENREF_16)) |
|  |  | Sand steenbras | Lithognathus mormyrus | 0.53 | 0.97 | ([Pajuelo et al., 2002](#_ENREF_89)) |
|  |  | Roughhead grenadier | Macrourus berglax | 0.87 | 0.93 | ([Rodríguez-Marín et al., 2002](#_ENREF_102)) |
|  |  | Two-banded seabream | Diplodus vulgaris | 0.45 | 0.67 | ([Pajuelo and Lorenzo, 2003](#_ENREF_88)) |
|  |  | Roughhead grenadier | Macrourus berglax | 0.91 | 0.62 | ([Rodríguez-Marín et al., 2002](#_ENREF_102)) |
|  |  | Cadenat's rockfish | Scorpaena loppei | 0.35 | 0.55 | ([Ordines et al., 2012](#_ENREF_85)) |
|  |  | West coast steenbras | Lithognathus aureti | 0.87 | 0.36 | ([Holtzhausen and Kirchner, 2001](#_ENREF_39)) |
|  |  | Antarctic plunderfish | Dolloidraco longedorsalis | 0.30 | 0.23 | ([Morales-Nin et al., 2000](#_ENREF_63)) |
|  |  |  |  |  |  |  |
| Skeletochronology | Mammal | Grey mouse lemur | Microcebus murin us | 1.00 | 4.80 | ([Castanet et al., 2004](#_ENREF_11)) |
|  | Reptile | Green sea turtle | Chelonia mydas | 1.00 | Inf | ([Snover et al., 2011](#_ENREF_107)) |
|  |  | Nile monitor | Varanus niloticus | 1.00 | Inf | ([de Buffrénil and Castanet, 2000](#_ENREF_15)) |
|  |  | Kemp's ridley sea turtles | Lepidochelys kempii | 0.93 | 2.20 | ([Snover and Hohn, 2004](#_ENREF_106)) |
|  |  | Freshwater crocodile | Crocodylus johnstoni | 0.99 | 1.51 | ([Tucker, 1997](#_ENREF_111)) |
|  |  | Arizona Tiger Salamander | Ambystoma tigrinum nebulosum | 0.74 | 0.29 | ([Eden et al., 2007](#_ENREF_18)) |
|  |  | Green turtles | Chelonia mydas | 0.00 | 0.00 | ([Bjorndal et al., 1998](#_ENREF_6)) |
|  | Amphibian | European tree frog | Hyla arborea | 1.00 | Inf | ([Friedl and Klump, 1997](#_ENREF_21)) |

Note: OW, otolith weight; OL, otolith length; OH, otolith height; OB, otolith breath. Inf, infinite $|\beta/\sigma|$for $\sigma$ = 0, i.e. no error in age determination.

**References**

ALLSOPP, R. C., VAZIRI, H., PATTERSON, C., GOLDSTEIN, S., YOUNGLAI, E. V., FUTCHER, A. B., GREIDER, C. W. & HARLEY, C. B. 1992. Telomere length predicts replicative capacity of human fibroblasts. *Proceedings of the National Academy of Sciences of the United States of America,* 89**,** 10114-10118.

ANGELIER, F., VLECK, C. M., HOLBERTON, R. L. & MARRA, P. P. 2013. Telomere length, non-breeding habitat and return rate in male American redstarts. *Functional Ecology,* 27**,** 342-350.

ARGYLE, D., ELLSMORE, V., GAULT, E. A., MUNRO, A. F. & NASIR, L. 2003. Equine telomeres and telomerase in cellular immortalisation and ageing. *Mechanisms of Ageing and Development,* 124**,** 759-764.

BEKAERT, B., KAMALANDUA, A., ZAPICO, S. C., VAN DE VOORDE, W. & DECORTE, R. 2015. Improved age determination of blood and teeth samples using a selected set of DNA methylation markers. *Epigenetics,* 10**,** 922-930.

BIZE, P., CRISCUOLO, F., METCALFE, N. B., NASIR, L. & MONAGHAN, P. 2009. Telomere dynamics rather than age predict life expectancy in the wild. *PROCEEDINGS OF THE ROYAL SOCIETY B-BIOLOGICAL SCIENCES,* 276 1679-1683.

BJORNDAL, K. A., BOLTEN, A. B., BENNETT, R. A., JACOBSON, E. R., WRONSKI, T. J., VALESKI, J. J. & ELIAZAR, P. J. 1998. Age and growth in sea turtles: limitations of skeletochronology for demographic studies. *Copeia***,** 23-30.

BOCKLANDT, S., LIN, W., SEHL, M. E., SÁNCHEZ, F. J., SINSHEIMER, J. S., HORVATH, S. & VILAIN, E. 2011. Epigenetic Predictor of Age. *PLoS ONE,* 6**,** e14821.

BRONIKOWSKI, A. M. 2008. The evolution of aging phenotypes in snakes: A review and synthesis with new data. *Age,* 30**,** 169-176.

BROUWER, S. L. & GRIFFITHS, M. H. 2004. Age and growth of *Argyrozona argyrozona* (Pisces: Sparidae) in a marine protected area: an evaluation of methods based on whole otoliths, sectioned otoliths and mark-recapture. *Fisheries Research,* 67**,** 1-12.

CAPPO, M., EDEN, P., NEWMAN, S. J. & ROBERTSON, S. 2000. A new approach to validation of periodicity and timing of opaque zone formation in the otoliths of eleven species of *Lutjanus* from the central Great Barrier Reef. *Fishery Bulletin,* 98**,** 474-488.

CASTANET, J., CROCI, S., AUJARD, F., PERRET, M., CUBO, J. & DE MARGERIE, E. 2004. Lines of arrested growth in bone and age estimation in a small primate: Microcebus murinus. *Journal of Zoology,* 263**,** 31-39.

CHO, S., GE, J., SEO, S. B., KIM, K., LEE, H. Y. & LEE, S. D. 2014. Age estimation via quantification of signal-joint T cell receptor excision circles in Koreans. *Legal Medicine,* 16**,** 135-138.

CHRISTENSEN, B. C., HOUSEMAN, E. A., MARSIT, C. J., ZHENG, S., WRENSCH, M. R., WIEMELS, J. L., NELSON, H. H., KARAGAS, M. R., PADBURY, J. F., BUENO, R., SUGARBAKER, D. J., YEH, R.-F., WIENCKE, J. K. & KELSEY, K. T. 2009. Aging and Environmental Exposures Alter Tissue-Specific DNA Methylation Dependent upon CpG Island Context. *PLoS Genet,* 5**,** e1000602.

D'ONGHIA, G., SION, L., MAIORANO, P., MYTILINEOU, C., DALESSANDRO, S., CARLUCCI, R. & DESANTIS, S. 2006. Population biology and life strategies of *Chlorophthalmus agassizii* Bonaparte, 1840 (Pisces: Osteichthyes) in the Mediterranean Sea. *Marine Biology,* 149**,** 435-446.

DE BUFFRÉNIL, V. & CASTANET, J. 2000. Age estimation by skeletochronology in the Nile monitor (Varanus niloticus), a highly exploited species. *Journal of Herpetology***,** 414-424.

DOMÍNGUEZ-SEOANE, R., PAJUELO, J. G., LORENZO, J. M. & RAMOS, A. G. 2006. Age and growth of the sharpsnout seabream *Diplodus puntazzo* (Cetti, 1777) inhabiting the Canarian archipelago, estimated by reading otoliths and by backcalculation. *Fisheries Research,* 81**,** 142-148.

DOUEK, D. C., MCFARLAND, R. D., KEISER, P. H., GAGE, E. A., MASSEY, J. M., HAYNES, B. F., POLIS, M. A., HAASE, A. T., FEINBERG, M. B., SULLIVAN, J. L., JAMIESON, B. D., ZACK, J. A., PICKER, L. J. & KOUP, R. A. 1998. Changes in thymic function with age and during the treatment of HIV infection. *Nature,* 396**,** 690-695.

EDEN, C. J., WHITEMAN, H. H., DUOBINIS-GRAY, L. & WISSINGER, S. A. 2007. Accuracy assessment of skeletochronology in the Arizona tiger salamander (Ambystoma tigrinum nebulosum). *Copeia,* 2007**,** 471-477.

FOOTE, C. G. 2008. *Avian telomere dynamics.* PhD thesis, University of Glasgow.

FRANCIS, R. I. C. C., PAUL, L. J. & MULLIGAN, K. P. 1992. Ageing of adult snapper (*Pagrus auratus*) from otolith annual ring counts: validation by tagging and oxytetracycline injection. *Australian Journal of Marine & Freshwater Research,* 43**,** 1069-1089.

FRIEDL, T. W. & KLUMP, G. M. 1997. Some aspects of population biology in the European treefrog, Hyla arborea. *Herpetologica***,** 321-330.

FU, S. J., FAN, C. C., SONG, H. W. & WEI, F. Q. 1995. Age estimation using a modified HPLC determination of ratio of aspartic acid in dentin. *Forensic Science International,* 73**,** 35-40.

FUJII, N., TAKEMOTO, L. J., MOMOSE, Y., MATSUMOTO, S., HIROKI, K. & AKABOSHI, M. 1999. Formation of four isomers at the Asp-151 residue of aged human αA-crystallin by natural aging. *Biochemical and Biophysical Research Communications,* 265**,** 746-751.

FURUGORI, E., HIRAYAMA, R., NAKAMURA, K. I., KAMMORI, M., ESAKI, Y. & TAKUBO, K. 2000. Telomere shortening in gastric carcinoma with aging despite telomerase activation. *Journal of Cancer Research and Clinical Oncology,* 126**,** 481-485.

GARDE, E., FRIE, A. K., DUNSHEA, G., HANSEN, S. H., KOVACS, K. M. & LYDERSEN, C. 2010. Harp seal ageing techniques-teeth, aspartic acid racemization, and telomere sequence analysis. *Journal of Mammalogy,* 91**,** 1365-1374.

GARDE, E., HEIDE-JORGENSEN, M. P., HANSEN, S. H., NACHMAN, G. & FORCHHAMMER, M. C. 2007. Age-specific growth and remarkable longevity in narwhals (*Monodon monoceros*) from West Greenland as estimated by aspartic acid racemization. *Journal of Mammalogy,* 88**,** 49-58.

GH, E., SAVARI, A., KOCHANIAN, P. & MOTLAGH A, T. 2012. Age, growth and length at first maturity of Otolithes ruber in the Northwestern part of the Persian Gulf, based on age estimation using otolith. *Iran. J. Fish. Sci,* 11**,** 13-27.

GOOLEY, G. J. 1992. Validation of the use of otoliths to determine the age and growth of Murray cod, *Maccullochella peelii* (Mitchell) (Percichthyidae), in Lake Charlegrark, western Victoria. *Australian Journal of Marine & Freshwater Research,* 43**,** 1091-1102.

HALL, M. E., NASIR, L., DAUNT, F., GAULT, E. A., CROXALL, J. P., WANLESS, S. & MONAGHAN, P. 2004. Telomere loss in relation to age and early environment in long-lived birds. *PROCEEDINGS OF THE ROYAL SOCIETY B-BIOLOGICAL SCIENCES,* 271**,** 1571-1576.

HANNUM, G., GUINNEY, J., ZHAO, L., ZHANG, L., HUGHES, G., SADDA, S., KLOTZLE, B., BIBIKOVA, M., FAN, J.-B., GAO, Y., DECONDE, R., CHEN, M., RAJAPAKSE, I., FRIEND, S., IDEKER, T. & ZHANG, K. 2013. Genome-wide Methylation Profiles Reveal Quantitative Views of Human Aging Rates. *Molecular Cell,* 49**,** 359-367.

HASTIE, N. D., DEMPSTER, M., DUNLOP, M. G., THOMPSON, A. M., GREEN, D. K. & ALLSHIRE, R. C. 1990. Telomere reduction in human colorectal carcinoma and with ageing. *Nature,* 346**,** 866-868.

HATAKEYAMA, H., NAKAMURA, K. I., IZUMIYAMA-SHIMOMURA, N., ISHII, A., TSUCHIDA, S., TAKUBO, K. & ISHIKAWA, N. 2008. The teleost *Oryzias latipes* shows telomere shortening with age despite considerable telomerase activity throughout life. *Mechanisms of Ageing and Development,* 129**,** 550-557.

HATASE, H., SUDO, R., WATANABE, K. K., KASUGAI, T., SAITO, T., OKAMOTO, H., UCHIDA, I. & TSUKAMOTO, K. 2008. Shorter telomere length with age in the loggerhead turtle: A new hope for live sea turtle age estimation. *Genes and Genetic Systems,* 83**,** 423-426.

HAUSSMANN, M. F. & MAUCK, R. A. 2008. New strategies for telomere-based age estimation. *Molecular Ecology Resources***,** 264–274.

HAUSSMANN, M. F. & VLECK, C. M. 2002. Telomere length provides a new technique for aging animals. *Oecologia***,** 325-328.

HAUSSMANN, M. F., VLECK, C. M. & NISBET, I. C. T. 2003a. Calibrating the telomere clock in common terns, *Sterna hirundo*. *Experimental Gerontology,* 38**,** 787-789.

HAUSSMANN, M. F., WINKLER, D. W., O'REILLY, K. M., HUNTINGTON, C. E., NISBET, I. C. T. & VLECK, C. M. 2003b. Telomeres shorten more slowly in long-lived birds and mammals than in short-lived ones. *Proceedings of the Royal Society B-Biological Sciences,* 270**,** 1387-1392.

HELFMAN, P. M. & BADA, J. L. 1976. Aspartic acid racemisation in dentine as a measure of ageing. *Nature,* 262**,** 279-281.

HOLTZHAUSEN, J. A. & KIRCHNER, C. H. 2001. Age and growth of two populations of West Coast steenbras *Lithognathus aureti* in Namibian waters, based on otolith readings and mark-recapture data. *South African Journal of Marine Science***,** 169-179.

HORN, T., GEMMELL, N. J., ROBERTSON, B. C. & BRIDGES, C. R. 2008. Telomere length change in European sea bass (*Dicentrarchus labrax*). *Australian Journal of Zoology,* 56**,** 207-210.

HORN, T., ROBERTSON, B. C., WILL, M., EASON, D. K., ELLIOTT, G. P. & GEMMELL, N. J. 2011. Inheritance of telomere length in a bird. *PLoS ONE,* 6**,** e17199.

HORVATH, S. 2013. DNA methylation age of human tissues and cell types. *Genome biology,* 14**,** R115.

HORVATH, S., ERHART, W., BROSCH, M., AMMERPOHL, O., VON SCHÖNFELS, W., AHRENS, M., HEITS, N., BELL, J. T., TSAI, P.-C. & SPECTOR, T. D. 2014. Obesity accelerates epigenetic aging of human liver. *Proceedings of the National Academy of Sciences,* 111**,** 15538-15543.

HUNTER, S. A. 1989. *Aspartic acid racemization in tendons as an indication of age in three avian species.* PhD thesis, Southern Illinois University at Carbondale.

ITO, G., YOSHIMURA, K. & MOMOI, Y. 2015. Gene analysis of signal-joint T cell receptor excision circles and their relationship to age in dogs. *Veterinary Immunology and Immunopathology,* 166**,** 1-7.

IZZO, C. 2010. *Patterns of telomere length change with age in aquatic vertebrates and the phylogenetic distribution of the pattern among jawed vertebrates.* PhD thesis, University of Adelaide.

JUOLA, F. A., HAUSSMANN, M. F., DEARBORN, D. C. & VLECK, C. M. 2006. Telomere shortening in a long-lived marine bird: cross-sectional analysis and test of an aging tool. *Auk,* 123**,** 775-783.

KANG, M. K., SWEE, J., KIM, R. H., BALUDA, M. A. & PARK, N.-H. 2002. The telomeric length and heterogeneity decrease with age in normal human oral keratinocytes. *Mechanisms of Ageing and Development,* 123**,** 585-592.

KATEPALLI, M. P., ADAMS, A. A., LEAR, T. L. & HOROHOV, D. W. 2008. The effect of age and telomere length on immune function in the horse. *Developmental & Comparative Immunology,* 32**,** 1409-1415.

KIRCHNER, C. H. & VOGES, S. F. 1999. Growth of Namibian silver kob *Argyrosomus inodorus* based on otoliths and mark-recapture data. *South African Journal of Marine Science***,** 201-209.

KOCH, C. M. & WAGNER, W. 2011. Epigenetic-aging-signature to determine age in different tissues. *Aging (Albany NY),* 3**,** 1018.

LAHNERT, P. 2005. An improved method for determining telomere length and its use in assessing age in blood and saliva. *Gerontology,* 51**,** 352-356.

LEE, H. Y., JUNG, S.-E., OH, Y. N., CHOI, A., YANG, W. I. & SHIN, K.-J. 2015. Epigenetic age signatures in the forensically relevant body fluid of semen: a preliminary study. *Forensic science international. Genetics,* 19**,** 28-34.

LEE, W. W., NAM, K. H., TERAO, K. & YOSHIKAWA, Y. 2002. Age-related telomere length dynamics in peripheral blood mononuclear cells of healthy cynomolgus monkeys measured by Flow FISH. *Immunology,* 105**,** 458-465.

LINDSEY, J., MCGILL, N. I., LINDSEY, L. A., GREEN, D. K. & COOKE, H. J. 1991. In vivo loss of telomeric repeats with age in humans. *Mutation Research,* 256**,** 45-48.

MAEGAWA, S., HINKAL, G., KIM, H. S., SHEN, L., ZHANG, L., ZHANG, J., ZHANG, N., LIANG, S., DONEHOWER, L. A. & ISSA, J.-P. J. 2010. Widespread and tissue specific age-related DNA methylation changes in mice. *Genome research,* 20**,** 332-340.

MAN, E. H., SANDHOUSE, M. E., BURG, J. & FISHER, G. H. 1983. Accumulation of D-aspartic acid with age in the human brain. *Science,* 220**,** 1407-8.

MANOOCH III, C. S. & POTTS, J. C. 1997. Age, growth and mortality of greater amberjack from the southeastern United States. *Fisheries Research,* 30**,** 229-240.

MAROUDAS, A., BAYLISS, M. T., UCHITEL-KAUSHANSKY, N., SCHNEIDERMAN, R. & GILAV, E. 1998. Aggrecan turnover in human articular cartilage: use of aspartic acid racemization as a marker of molecular age. *Archives of Biochemistry and Biophysics,* 350**,** 61-71.

MASTERS, P. M., BADA, J. L. & ZIGLER, J. S. 1977. Aspartic-acid racemization in human lens during aging and in cataract formation. *Nature,* 268**,** 71-73.

MELK, A., RAMASSAR, V., HELMS, L. M. H., MOORE, R., RAYNER, D., SOLEZ, K. & HALLORAN, P. F. 2000. Telomere shortening in kidneys with age. *Journal of the American Society of Nephrology,* 11**,** 444-453.

MIYASHITA, N., SHIGA, K., YONAI, M., KANEYAMA, K., KOBAYASHI, S., KOJIMA, T., GOTO, Y., KISHI, M., ASO, H., SUZUKI, T., SAKAGUCHI, M. & NAGAI, T. 2002. Remarkable differences in telomere lengths among cloned cattle derived from different cell types. *Biology of Reproduction,* 66**,** 1649-1655.

MORALES-NIN, B., MORANTA, J. & BALGUERIAS, E. 2000. Growth and age validation in high-Antarctic fish. *Polar Biology,* 23**,** 626-634.

MUG-VILLANUEVA, M., GALLUCCI, V. F. & LAI, H.-L. 1994. Age determination of corvina reina (*Cynoscion albus*) in the Gulf of Nicoya, Costa Rica, based on examination and analysis of hyaline zones, morphology and microstructure of otoliths. *Journal of Fish Biology,* 45**,** 177-191.

NASIR, L., DEVLIN, P., MCKEVITT, T., RUTTEMAN, G. & ARGYLE, D. J. 2001. Telomere lengths and telomerase activity in dog tissues: A potential model system to study human telomere and telomerase biology. *Neoplasia,* 3**,** 351-359.

NEWMAN, S. J. 2002a. Age, growth, mortality and population characteristics of the pearl perch, *Glaucosoma buergeri* Richardson 1845, from deeper continental shelf waters off the Pilbara coast of north-western Australia. *Journal of Applied Ichthyology,* 18**,** 95-101.

NEWMAN, S. J. 2002b. Growth rate, age determination, natural mortality and production potential of the scarlet seaperch, *Lutjanus malabaricus* Schneider 1801, off the Pilbara coast of north-western Australia. *Fisheries Research,* 58**,** 215-225.

NEWMAN, S. J., CAPPO, M. & WILLIAMS, D. M. 2000a. Age, growth and mortality of the stripey, *Lutjanus carponotatus* (Richardson) and the brown-stripe snapper, *L. vitta* (Quoy and Gaimard) from the central Great Barrier Reef, Australia. *Fisheries Research,* 48**,** 263-275.

NEWMAN, S. J., CAPPO, M. & WILLIAMS, D. M. 2000b. Age, growth, mortality rates and corresponding yield estimates using otoliths of the tropical red snappers, *Lutjanus erythropterus*, *L. malabaricus* and *L. sebae*, from the central Great Barrier Reef. *Fisheries Research,* 48**,** 1-14.

NEWMAN, S. J. & DUNK, I. J. 2002. Growth, age validation, mortality, and other population characteristics of the red emperor snapper, *Lutjanus sebae* (Cuvier, 1828), off the Kimberley coast of north-western Australia. *ESTUARINE COASTAL AND SHELF SCIENCE,* 55**,** 67-80.

NEWMAN, S. J., WILLIAMS, D. M. & RUSS, G. R. 1996. Age validation, growth and mortality rates of the tropical snappers (Pisces: Lutjanidae) *Lutjanus adetii* (Castelnau, 1873) and *L. quinquelineatus* (Bloch, 1790) from the central Great Barrier Reef, Australia. *Marine and Freshwater Research,* 47**,** 575-584.

OGINO, T., OGINO, H. & NAGY, B. 1985. Application of aspartic acid racemization to forensic odontology: Post mortem designation of age at death. *Forensic Science International,* 29**,** 259-267.

OHTANI, S. 1994. Age estimation by aspartic acid racemization in dentin of deciduous teeth. *Forensic Science International,* 68**,** 77-82.

OHTANI, S. 1995. Studies on age estimation using racemization of aspartic acid in cementum. *Journal of Forensic Sciences,* 40**,** 805-7.

OHTANI, S., MATSUSHIMA, Y., KABAYASHI, Y. & KISHI, K. 1998. Evaluation of aspartic acid racemization ratios in the human femur for age estimation. *Journal of forensic sciences,* 43**,** 949-953.

OHTANI, S., MATSUSHIMA, Y., KOBAYASHI, Y. & YAMAMOTO, T. 2002. Age estimation by measuring the racemization of aspartic acid from total amino acid content of several types of bone and rib cartilage: A preliminary account. *Journal of Forensic Sciences,* 47**,** 32-36.

OHTANI, S., SUGIMOTO, H., SUGENO, H., YAMAMOTO, S. & YAMAMOTO, K. 1995. Racemization of aspartic-acid in human cementum with age. *Archives of Oral Biology,* 40**,** 91-95.

OHTANI, S., YAMADA, Y., YAMAMOTO, T., ARANY, S., GONMORI, K. & YOSHIOKA, N. 2004. Comparison of age estimated from degree of racemization of aspartic acid, glutamic acid and alanine in the femur. *Journal of Forensic Sciences,* 49**,** 441-445.

OHTANI, S. & YAMAMOTO, K. 1987. Age estimation using the racemization of aspartic acid on human dentin. *Nihon Hoigaku Zasshi,* 41**,** 181-90.

OHTANI, S. & YAMAMOTO, K. 1991. Age estimation using the racemization of amino acid in human dentin. *Journal of Forensic Sciences,* 36**,** 792-800.

OHTANI, S. & YAMAMOTO, K. 1992. Estimation of age from a tooth by means of racemization of an amino acid, especially aspartic acid--comparison of enamel and dentin. *Journal of Forensic Sciences,* 37**,** 1061-7.

OHTANI, S. & YAMAMOTO, T. 2010. Age estimation by amino acid racemization in human teeth. *Journal of Forensic Sciences,* 55**,** 1630-1633.

OHTANI, S. & YAMAMOTO, T. 2011. Comparison of age estimation in Japanese and Scandinavian teeth using amino acid racemization. *Journal of Forensic Sciences,* 56**,** 244-247.

OLSSON, M., PAULINY, A., WAPSTRA, E., ULLER, T., SCHWARTZ, T. & BLOMQVIST, D. 2011. Sex differences in sand lizard telomere inheritance: Paternal epigenetic effects increases telomere heritability and offspring survival. *PLoS ONE,* 6**,** e17473.

ORDINES, F., VALLS, M. & GOURAGUINE, A. 2012. Biology, feeding, and habitat preferences of Cadenat's rockfish, *Scorpaena loppei* (Actinopterygii: Scorpaeniformes: Scorpaenidae), in the Balearic Islands (western mediterranean). *Acta Ichthyologica et Piscatoria,* 42**,** 21-30.

OU, X.-L., GAO, J., WANG, H., WANG, H.-S., LU, H.-L. & SUN, H.-Y. 2012. Predicting Human Age with Bloodstains by sjTREC Quantification. *Plos One,* 7**,** e42412.

OU, X., ZHAO, H., SUN, H., YANG, Z., XIE, B., SHI, Y. & WU, X. 2011. Detection and quantification of the age-related sjTREC decline in human peripheral blood. *International Journal of Legal Medicine,* 125**,** 603-608.

PAJUELO, J. G. & LORENZO, J. M. 2003. The growth of the common two-banded seabream, *Diplodus vulgaris* (Teleostei, Sparidae), in Canarian waters, estimated by reading otoliths and by back-calculation. *Journal of Applied Ichthyology,* 19**,** 79-83.

PAJUELO, J. G., LORENZO, J. M., MÉNDEZ, M., COCA, J. & RAMOS, A. G. 2002. Determination of age and growth of the striped seabream *Lithognathus mormyrus* (Sparidae) in the Canarian archipelago by otolith readings and backcalculation. *Scientia Marina,* 66**,** 27-32.

PAULINY, A., WAGNER, R. H., AUGUSTIN, J., SZÉP, T. & BLOMQVIST, D. 2006. Age-independent telomere length predicts fitness in two bird species. *Molecular Ecology,* 15**,** 1681-1687.

PFEIFFER, H., MÖRNSTAD, H. & TEIVENS, A. 1995a. Estimation of chronologic age using the aspartic acid racemization method. I. On human rib cartilage. *International journal of legal medicine,* 108**,** 19-23.

PFEIFFER, H., MÖRNSTAD, H. & TEIVENS, A. 1995b. Estimation of chronologic age using the aspartic acid racemization method. II. On human cortical bone. *International Journal of Legal Medicine,* 108**,** 24-26.

POLANOWSKI, A. M., ROBBINS, J., CHANDLER, D. & JARMAN, S. N. 2014. Epigenetic estimation of age in humpback whales. *Molecular Ecology Resources,* 14**,** 976-987.

POTTS, J. C., MANOOCH III, C. S. & VAUGHAN, D. S. 1998. Age and growth of vermilion snapper from the Southeastern United States. *Transactions of the American Fisheries Society,* 127**,** 787-795.

QU, D.-Y., DENG, S.-J., GE, Y.-Y., CHEN, S. & OU, X.-L. 2013. Age estimation using content of sjTREC in human peripheral blood. *Fa yi xue za zhi,* 29**,** 256-272.

RAJKUMARI, S., NIRMAL, M., SUNIL, P. M. & SMITH, A. A. 2013. Estimation of age using aspartic acid racemisation in human dentin in Indian population. *Forensic Science International,* 228**,** 38-41.

RITZ-TIMME, S., LAUMEIER, I. & COLLINS, M. 2003. Age estimation based on aspartic acid racemization in elastin from the yellow ligaments. *International Journal of Legal Medicine,* 117**,** 96-101.

RITZ, S., SCHÜTZ, H. W. & PEPER, C. 1993. Postmortem estimation of age at death based on aspartic acid racemization in dentin: Its applicability for root dentin. *International Journal of Legal Medicine,* 105**,** 289-293.

RITZ, S., SCHÜTZ, H. W. & SCHWARZER, B. 1990. The extent of aspartic acid racemization in dentin: a possible method for a more accurate determination of age at death? *Zeitschrift für Rechtsmedizin,* 103**,** 457-462.

RITZ, S., TURZYNSKI, A. & SCHÜTZ, H. W. 1994. Estimation of age at death based on aspartic acid racemization in noncollagenous bone proteins. *Forensic Science International,* 69**,** 149-159.

RITZ, S., TURZYNSKI, A., SCHÜTZ, H. W., HOLLMANN, A. & ROCHHOLZ, G. 1996. Identification of osteocalcin as a permanent aging constituent of the bone matrix: basis for an accurate age at death determination. *Forensic Science International,* 77**,** 13-26.

RODRÍGUEZ-MARÍN, E., RUIZ, M. & SARASUA, A. 2002. Validation of roughhead grenadier (*Macrourus berglax*) otolith reading. *Journal of Applied Ichthyology,* 18**,** 70-80.

SHAPIRO, S. D., ENDICOTT, S. K., PROVINCE, M. A., PIERCE, J. A. & CAMPBELL, E. J. 1991. Marked longevity of human lung parenchymal elastic fibers deduced from prevalence of D-aspartate and nuclear weapons-related radiocarbon. *Journal of Clinical Investigation,* 87**,** 1828-34.

SHIELS, P. G., KIND, A. J., CAMPBELL, K. H. S., WADDINGTON, D., WILMUT, I., COLMAN, A. & SCHNIEKE, A. E. 1999. Analysis of telomere lengths in cloned sheep. *Nature,* 399**,** 316-317.

SHIMOYAMA, A. & HARADA, K. 1984. An age-determination of an ancient burial mound man by apparent racemization reaction of aspartic-acid in tooth dentin. *Chemistry Letters***,** 1661-1664.

SNOVER, M. L. & HOHN, A. A. 2004. Validation and interpretation of annual skeletal marks in loggerhead (Caretta caretta) and Kemp’s ridley (Lepidochelys kempii) sea turtles. *Fishery Bulletin,* 102**,** 682-692.

SNOVER, M. L., HOHN, A. A., GOSHE, L. R. & BALAZS, G. H. 2011. Validation of annual skeletal marks in green sea turtles Chelonia mydas using tetracycline labeling. *Aquatic Biology,* 12**,** 197-204.

TAKASAKI, T., TSUJI, A., IKEDA, N. & OHISHI, M. 2003. Age estimation in dental pulp DNA based on human telomere shortening. *International Journal of Legal Medicine,* 117**,** 232-234.

TSUI, J. C. Y. 2005. *Evaluation of telomere length as an age-marker in marine teleosts.* MSc thesis, The University of Hong Kong.

TSUJI, A., ISHIKO, A., TAKASAKI, T. & IKEDA, N. 2002. Estimating age of humans based on telomere shortening. *Forensic Science International,* 126**,** 197-199.

TUCKER, A. D. 1997. Validation of skeletochronology to determine age of freshwater crocodiles (Crocodylus johnstoni). *Marine and Freshwater Research,* 48**,** 343-351.

UJVARI, B. & MADSEN, T. 2009. Short telomeres in hatchling snakes: erythrocyte telomere dynamics and longevity in tropical pythons. *PloS One,* 4**,** e7493.

UNRYN, B. M., COOK, L. S. & RIABOWOL, K. T. 2005. Paternal age is positively linked to telomere length of children. *Aging Cell,* 4**,** 97-101.

VAN DEN OETELAAR, P. J. & HOENDERS, H. J. 1989. Racemization of aspartyl residues in proteins from normal and cataractous human lenses: an aging process without involvement in cataract formation. *Experimental Eye Research,* 48**,** 209-14.

VERZIJL, N., DEGROOT, J., THORPE, S. R., BANK, R. A., SHAW, J. N., LYONS, T. J., BIJLSMA, J. W. J., LAFEBER, F. P. J. G., BAYNES, J. W. & TEKOPPELE, J. M. 2000. Effect of collagen turnover on the accumulation of advanced glycation end products. *Journal of Biological Chemistry,* 275**,** 39027-39031.

WEIDNER, C. I., LIN, Q., KOCH, C. M., EISELE, L., BEIER, F., ZIEGLER, P., BAUERSCHLAG, D. O., JÖCKEL, K.-H., ERBEL, R. & MÜHLEISEN, T. W. 2014. Aging of blood can be tracked by DNA methylation changes at just three CpG sites. *Genome biology,* 15**,** R24.

WHITE, D. B. & PALMER, S. M. 2004. Age, growth, and reproduction of the red snapper, *Lutjanus campechanus*, from the atlantic waters of the southeastern U.S. *Bulletin of Marine Science,* 75**,** 335-360.

WIEMANN, S. U., SATYANARAYANA, A., TSAHURIDU, M., TILLMANN, H. L., ZENDER, L., KLEMPNAUER, J., FLEMMING, P., FRANCO, S., BLASCO, M. A., MANNS, M. P. & RUDOLPH, K. L. 2002. Hepatocyte telomere shortening and senescence are general markers of human liver cirrhosis. *FASEB JOURNAL,* 16**,** 935-942.

YANG, L., SUWA, T., WRIGHT, W. E., SHAY, J. W. & HORNSBY, P. J. 2001. Telomere shortening and decline in replicative potential as a function of donor age in human adrenocortical cells. *Mechanisms of Ageing and Development,* 122**,** 1685-1694.

YAZAWA, M., OKUDA, M., SETOGUCHI, A., IWABUCHI, S., NISHIMURA, R., SASAKI, N., MASUDA, K., OHNO, K. & TSUJIMOTO, H. 2001. Telomere length and telomerase activity in canine mammary gland tumors. *American Journal of Veterinary Research,* 62**,** 1539-1543.

YOUNG, R. C., KITAYSKY, A. S., HAUSSMANN, M. F., DESCAMPS, S., ORBEN, R. A., ELLIOTT, K. H. & GASTON, A. J. 2013. Age, sex, and telomere dynamics in a long-lived seabird with male-biased parental care. *PLoS ONE,* 8**,** e74931.

ZBIEC-PIEKARSKA, R., SPOLNICKA, M., KUPIEC, T., MAKOWSKA, Z., SPAS, A., PARYS-PROSZEK, A., KUCHARCZYK, K., PLOSKI, R. & BRANICKI, W. 2015. Examination of DNA methylation status of the ELOVL2 marker may be useful for human age prediction in forensic science. *Forensic Science International-Genetics,* 14**,** 161-167.

ZUBAKOV, D., LIU, F., VAN ZELM, M., VERMEULEN, J., OOSTRA, B., VAN DUIJN, C., DRIESSEN, G., VAN DONGEN, J., KAYSER, M. & LANGERAK, A. 2010. Estimating human age from T-cell DNA rearrangements. *Current Biology,* 20**,** R970-R971.
