# Supplementary material for: The adequacy of aging techniques in vertebrates for rapid estimation of population mortality rates from age distributions
Source: Ecol Evol. 2018 Dec 27;9(3):1394–402. doi: 10.1002/ece3.4854 (PMC6374686; doi:10.1002/ece3.4854)
Supplement: Supplementary file 5 [file ECE3-9-1394-s005.docx]

**Appendix S4. Example of R code to simulate data and calculate** $\frac{\boldsymbol{1}}{\boldsymbol{m}\sqrt{\boldsymbol{I}\left( \boldsymbol{m} \right)}}$**, the basic factor in the 95% error percentage plotted in Fig. 1.**

############### Parameters that will be used in the function #######################

# beta$\beta$ and sigma$\sigma$ is the slope and the standard deviation of the error in equation 19,

# respectively.

#$m$, assumed mortality rate.

# $c$, constant used in calculation of $K(\mu)$, discussed in Numerical Computation Fisher

# Information in Appendix S2.

# Construct a function called basic factor to calculate $\frac{1}{m\sqrt{I\left( m \right)}}$, the basic factor in

# 95% error percentage ($EP(95)$)in equation 44.

basic_factor <- function(beta = 1, sigma, m, c) {

# use $m$, beta$\beta$, sigma$\sigma$ to calculate proxy coefficient mu$\mu$

beta = abs(beta)

lambda = -log(1-m) # equation is specified in the line below equation

mu <- sigma*lambda/beta # equation is specified in the line above equation 22

if(mu > 8) {NA} else { # when mu $\mu$ > 8 the computation is unreliable and thus discarded

# integral part of equation 46

int10 <- function(x) (x + dnorm(x)/pnorm(x)) * dnorm(x + mu)

int1 <- integrate(int10, -mu - c, -mu + c)$value

# equation 46 to calculate $L\left( \mu\right)$, which is also the lower limit of $K\left( \mu\right)$

L <- Klow <- mu + int1

# right hand of equation 47, which is the upper limit of$K\left( \mu\right)$

Kup <- L + (1 + 1/c + 1/c^2) * dnorm(c)

# use the average of the upper and lower limit to represent $K\left( \mu\right)$ in equation 47

K <- (Kup + Klow)/2

Jmu <- 1/(mu^2) - 1- mu^2 + mu*K # second equation in equation 45

Im <- (sigma/beta/(1-m))^2*Jmu # equation 38

mIm <- 1/(sqrt(Im)*m) # $\frac{1}{m\sqrt{I\left( m \right)}}$, the basic factor in equation 44 and plotted in Fig. 1

# return $\frac{1}{m\sqrt{I\left( m \right)}}$ in equation 44

if(is.infinite(mIm)|is.nan(mIm)) {

NA # when mu $\mu$ > 8 the computation is unreliable and thus discarded

} else {

mIm

}

}

}
