# Supplementary material for: The adequacy of aging techniques in vertebrates for rapid estimation of population mortality rates from age distributions
Source: Ecol Evol. 2018 Dec 27;9(3):1394–402. doi: 10.1002/ece3.4854 (PMC6374686; doi:10.1002/ece3.4854)
Supplement: Supplementary file 6 [file ECE3-9-1394-s006.docx]

**Appendix S6:** **Key mathematical notation**

***General notation***

$F$: distribution functions

$f$: (probability) density functions

$\Phi(z)$: standard normal distribution function

$\varphi(z)$: standard normal density function

***Birth and survival related notation***

$S$*:* survival time

$F_{S}(s)$: the distribution function of survival time $S$

$f_{S}(s)$: the density function of survival time $S$

$T$*:* time of birth of a random individual from the population

$f_{T}(t)$: the density function of time of birth $T$

$\tau$: the maximum possible age an individual from the population can attain

***Linear regression related notation***

$X$: age proxy

$X_{1},\ldots,X_{n}$: independent and identically distributed copies of $X$

$x_{1},\ldots,x_{n}$: realization of the random variables $X_{1},\ldots,X_{n}$

$Y$: age

$f_{Y}(y)$: density function of the age of an individual at time 0

$g(y)$: regression function relating age proxy to age

$\alpha$: intercept for linear regression of age proxy against age

$\beta$: slope for linear regression of age proxy against age

$\sigma$: standard deviation of the error in the regression model

$\varepsilon$: standardized error in the regression model

$f_{\varepsilon}(z)$: density function for $\varepsilon$

$F_{\varepsilon}(z)$: distribution function for $\varepsilon$

$n$: sample size, i.e., the number of sampled individuals of which the age proxy is measured

***Mortality rate related notation***

$m$: mortality rate

$\lambda=-ln(1-m)$: rate parameter of exponential distribution

$\beta/\sigma$: the crucial indicator for the variation in estimated mortality rate

$\mu=\sigma\lambda/|\beta|$: proxy coefficient

$\hat{\mu}_{n}$: asymptotically efficient estimator of $\mu$

$I(m)$: Fisher information for mortality rate $m$

$J(\mu)$: Fisher information for $\mu$

$CR(95)$: 95% confidence range

$EP(95)$: theoretical 95% error percentage for mortality rate $m$

$EEP(95)$: empirical 95% error percentage for mortality rate $m$

$\hat{m}_{n}$: efficient estimator for mortality rate $m$

$\frac{\boldsymbol{1}}{\boldsymbol{m}\sqrt{\boldsymbol{I}\left( \boldsymbol{m} \right)}}$**:** the basic factor used in the calculation of 95% error percentage $EEP(95)$
